# Phage Delivery Strategies for Biocontrolling Human, Animal, and Plant Bacterial Infections: State of the Art

**DOI:** 10.3390/pharmaceutics16030374

**Published:** 2024-03-08

**Authors:** Marta M. D. C. Vila, Liliane M. N. Balcão, Victor M. Balcão

**Affiliations:** 1 VBlab—Laboratory of Bacterial Viruses, University of Sorocaba, Sorocaba 18023-000, SP, Brazil; lilianenavarro@gmail.com; 2 Department of Biology and CESAM, University of Aveiro, Campus Universitário de Santiago, 3810-193 Aveiro, Portugal

**Keywords:** phage delivery strategies, human, animal and plant bacterial infections, phage therapy, phage-based biocontrol

## Abstract

This review aims at presenting the main strategies that are currently available for the delivery of bacteriophages to combat bacterial infections in humans, animals, and plants. It can be seen that the main routes for phage delivery are topical, oral, systemic, and airways for humans. In animals, the topical and oral routes are the most used. To combat infections in plant species, spraying the plant’s phyllosphere or drenching the soil are the most commonly used methods. In both phage therapy and biocontrol using phages, very promising results have been obtained so far. However, more experiments are needed to establish forms of treatment and phage doses, among other parameters. Furthermore, in general, there is a lack of specific standards for the use of phages to combat bacterial infections.

## 1. Introduction

Bacteriophages (or, simply, phages) are defined as viruses that interact exclusively with bacterial cells (prokaryotes), infecting them. The bacteriophage virions hack into the inner metabolic machinery of bacterial cells, tricking them into replicating bacteriophage progeny instead of themselves. Without susceptible bacterial hosts nearby, phage virions crystallize, which leads to the following question: are phage virions living organisms or simply nature’s mechanical contraptions built from organic molecules, something between mechanical and living? The term bacteriophage is derived from “bacteria” and the Greek term “φαγεῖν” (*phagein*), which means “to devour” [1] Phages are structurally simple and consist of genetic material, either deoxyribonucleic acid (DNA) or ribonucleic acid (RNA), surrounded by a protein coat, or capsid. They are extremely diverse, representing the most abundant (living?) entities in the environment with an estimated total of 10^31^–10^32^ phage particles or more in the biosphere [2,3]. Bacteriophages are present wherever bacterial organisms thrive, including air, soil, river water, and the sea. Sewage is an endless source of bacteria, so too is sewage a source of bacteriophages [4]. Phages are obligate intracellular parasites of bacterial hosts with diverse life cycles including lytic, lysogenic, and pseudolysogenic cycles. In the lytic cycle, after the infection of a susceptible bacterial host cell, the phage starts producing new viral progeny and releases them by lysing the host via a coordinated two-enzyme system, viz. holins and endolysins. In the lysogenic cycle, the phage genome (in the form of a prophage) replicates with the host DNA, either integrating itself into the host’s chromosome or existing freely to form a long-lasting, stable coexistence with the host. Pseudolysogeny is the cycle where the phages neither lyse the host nor integrate their genome into the host’s genome to form a long-lasting stable connection [5].

**Figure 1:**
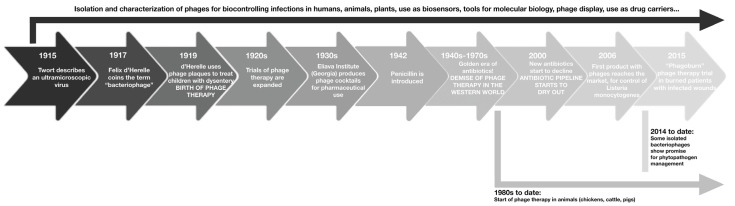
Timeline in the development of phages as potential therapeutic agents for bacterial infections.

Bacteriophages were discovered by Frederick Twort (1915) as unidentified molecules capable of inhibiting bacterial growth. He was the first to describe the potential existence of an “ultra-microscopic virus” that could harm bacteria [6]. In 1915, Félix D’Herelle investigated and isolated entities against dysentery bacilli. In 1917, the research of D’Herelle was presented to the Académie des Sciences, regarding “the invisible microbial antagonist of dysentery bacillus”, which was named “bacteriophage” [4,7]. However, only with the development of the electron microscope in the 1940s was the bacteriophage considered a single entity [4]. In 1923, George Eliava established the Georgia-based Eliava Institute with a focus on phage therapy. However, with the discovery of penicillin in 1928 and its high-scale production, the interest in phage therapy declined [8,9]. Since the discovery of bacteriophages, there has been interest in their use as human therapeutic agents due to their ability to modify or destroy bacteria. But, due to a lack of understanding of their mechanism of action, issues related to the stability of formulations, and the emergence of antibiotics, the interest in phage therapies was lost for decades in Western medicine [10]. Figure 1 illustrates the timeline in the development of phages as potential therapeutic agents for bacterial infections.

During the last decades, the emergence of multi-drug resistant (MDR) bacteria has been reported as a result of the common and frequent use of antibiotics in human and veterinary medicine, as well as in industry and agriculture [11]. Bacterial resistance to antibiotics has been known since the 1950s. In the following decades, bacteria developed many different mechanisms of resistance to antibiotics that protected them from the effects of these drugs, and, consequently, resistance to antibiotics has grown more and more widespread [6]. The scope of the antimicrobial resistance crisis exposes humanity to grave risks and may soon result in an alarming increase in the rates of infection by pathogenic bacteria that cause death and illness. Even though the acquisition and spread of resistance genes require time, the overuse and abuse of antibiotics greatly speed up the evolution of bacterial resistance [12].

Hence, the interest in bacteriophages has resurfaced once more [13,14]. The use of bacteriophages in the fight against bacterial infections promotes a non-antibiotic alternative with much greater specificity and without any harmful effects whatsoever upon the microbiota [1,3,6]. Phages target their specific bacterial hosts, replicate within their cells, and then destroy the host pathogen with high efficiency. Due to their ecological safety and specificity, phages are ideal tools for killing bacteria [15]. Therefore, the use of phages for combating bacteria may constitute a relevant weapon, in the short-term, for the battle against bacterial pathogens responsible for difficult-to-treat infections, considering the rapid loss of effectiveness of the current antimicrobial armamentarium that we are facing [12]. Briefly, it can be said that the use of phage therapy has some advantages, such as the following: (i) their ability to selectively target host bacteria, minimizing the disruption of the host’s native flora; (ii) bacteriophages also have the benefit of duplication at the site of infection; (iii) they are harmless to eukaryotic cells; (iv) bacteriophages can be adapted to infect resistant bacteria, if they develop resistance to them. Drawbacks include the following: (i) the need to identify the bacteria to be attacked beforehand; (ii) the need to employ a combination of bacteriophages (phage cocktail) to avoid or reduce the bacterial resistance; (iii) phages are self-limiting, meaning they need their hosts to be constantly expanding [2].

There are many potential therapeutic uses of phages to treat bacterial infections [16] and several studies have shown success in treatments involving bacteriophages [17]. It must be emphasized, however, that only lytic phages should be used in the fight against bacterial infections, not temperate ones, to avoid the possibility of the horizontal transfer of pathogenicity [18]. In addition, bacteriophages can be administered via different routes and using different formulations for the treatment of bacterial infections in humans, animals, and plants [19,20].

Growth-promoting antibiotics have been associated with animal production since the 1950s as a means to improve animal performance and promote health. However, the emergence of antibiotic resistance in bacterial pathogens identified as public health risks has led to a reduction in routine antibiotic supplementation for agricultural use and a complete ban in some parts of the world [21]. Hence, alternative methods for fighting infections, such as the use of bacteriophages, have started to be used in animal health, too. There is much research examining the efficacy of using phages as antibacterials, specifically in food-animal production, aiming to control bacterial diseases [22]. Particularly, phages have been studied for controlling bacterial diseases in poultry [7,11,23,24], pigs [22], calves [25], and fish [26]. Zoonotic pathogens associated with poultry and pigs, such as *Salmonella* spp., *E*. *coli*, *Campylobacter* spp., *Clostridium* spp., and *Listeria* spp., have emerged as resistant to several antibiotics, and the use of bacteriophages appears, thus, as an alternative potential treatment [27,28].

In agriculture, crop protection strategies based on beneficial microorganisms or naturally derived antimicrobial agents are being developed, aiming to reduce losses and impair advances from diseases caused by bacteria [29]. For plant crops, bacteriophage-based biocontrol has been used on several important bacterial plant pathogens, with very promising results [30]. In this way, over the years, many bacteriophages against many plant-pathogenic bacteria have been isolated from environmental sources, and diverse studies have shown that their use allows for the efficient management of disease development in both controlled (greenhouse) and open (field) conditions [31,32]. Nowadays, several phage-based biocontrol products have reached the market [30,33]. These products are cited as follows: Agriphage, a product designed for the control of bacterial spots of tomatoes and peppers (specific for *X*. *campestris* pv. *vesicatoria* or *P*. *syringae* pv. *tomato*) developed by the company Omnilytics; Erwiphage, for the control of fire blight of apple trees (specific for *Erwinia amylovora*), created by the Hungarian company, Enviroinvest; and Biolyse, developed for potato tubers for prevention of soft rot disease (specific against soft rot *Enterobacteriacea*) by the Scottish company, APS Biocontrol [30].

**Figure 2:**
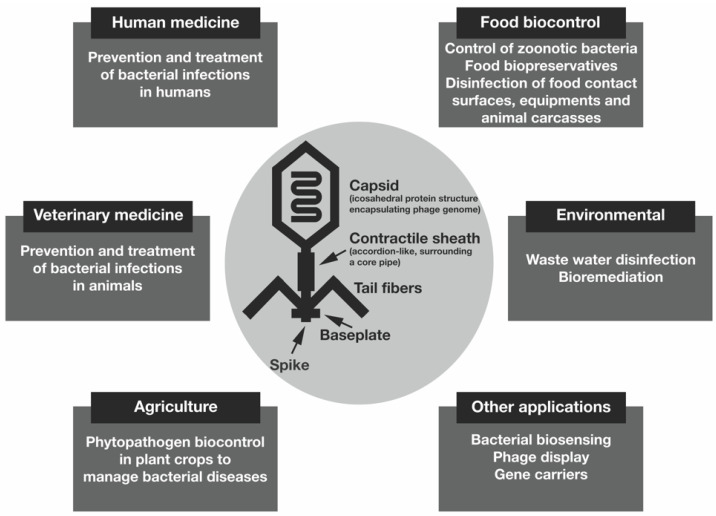
Potential applications of bacteriophages.

The potential uses of bacteriophages are very diverse, and their potential applications range from animal to human infections, gene delivery, food preservation, biocontrol of plant pathogens, bacterial biosensing devices, vaccines and gene carriers, among others, carried in different formulations [3,34,35]. Other potential applications of phages lie in the treatment of wastewater and bioremediation processes [36]. Figure 2 shows the potential (non-exhaustive) applications of bacteriophages.

The versatility of phages, paired with their apparent resilience and lack of metabolic machinery, allows them to be applied in various formulations (such as liquids and creams, and impregnated within solid matrices) either individually or combined with antibiotics [37].

**Figure 3:**
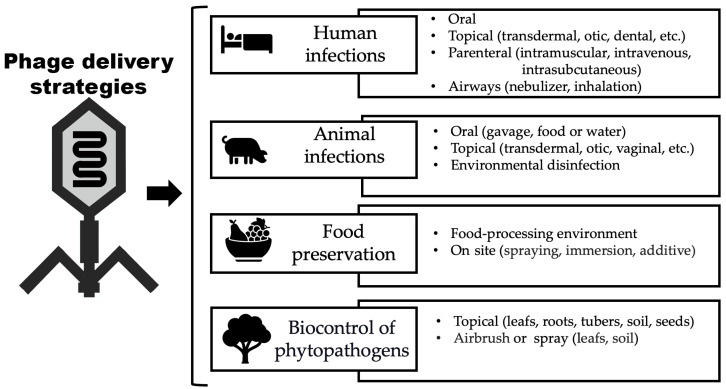
Ways in which phage virions can be delivered.

Phage delivery strategies are diverse and can be designed depending on the objective. Figure 3 illustrates different methods of phage delivery to combat human and animal infections, for food preservation, and the biocontrol of plants.

### 1.1. Phage Therapy and Phage-Based Biocontrol

The clinical administration of lytic phages directly to a patient (either human or animal) with the purpose of lysing the bacterial pathogen that is causing a clinically relevant infection is called phage therapy [38,39]. Biocontrol or biological control can be defined as the exploitation of living agents to fight pestilential organisms (pathogens, pests, and weeds) for diverse purposes, to provide human benefits [40]. The term “bacteriophage biocontrol”, or “phage biocontrol”, involves the pre- and post-harvest application of phages as well as the decontamination of food contact surfaces in food processing facilities [41].

Phage therapy and phage biocontrol represent interesting alternatives to eradicate multi-drug-resistant bacteria. There are, however, factors that limit their large-scale medical applicability, such as the loss of phage virion lytic activity under physiological conditions or in the environment [42]. In addition, the effectiveness of either bacteriophage therapy or phage biocontrol depends on factors such as the concentration of phage virions at the site of infection (dose) and their ability to reach their target bacterial cells and kill them. In addition, many studies have shown that the use of several different phages for the same bacterial host (phage cocktail) produces better results in preventing the emergence of mutant bacterial resistant strains [43].

The ability of phage virions to act only on extracellular bacteria together with the probability of interference by anti-phage antibodies in vivo are considered important limitations of bacteriophage therapy. Phage particles can be cleared by the immune system, and phage proteins are rapidly degraded by enzymes or inactivated by the low pH in the stomach [44,45]. Hence, to ensure the efficacy and effective delivery of therapeutic phages, carrier formulations have been under constant development [45,46,47]. For phage biocontrol applications the challenges are also great since studies in both the field and under laboratory conditions demonstrated that bacteriophages can be easily inactivated by exposure to high temperatures, high and low pH values, and sunlight (especially UV-A and UV-B radiation wavelengths of the spectrum) [31,48]. Depending on their intended place of action, strategies for the delivery of phage virions can differ significantly and, for successful phage therapy in the treatment of bacterial infections, the maintenance of both their activity and stability is of utmost importance. The use of phages presents one of the biggest challenges, related to the maintenance of their lytic viability, which depends on their biological properties as well as on environmental factors [49]. Many factors can affect the effectiveness of treatment in phage therapy, including (but not limited to) the phage/bacteria ratio (i.e., the multiplicity of infection (MOI)), the environmental conditions surrounding each phage virion, the emergence of bacterial resistance to the phage, the accessibility of the phage virions to the target bacterium, the initial phage dose, the moment of treatment for controlling the infection, the forms of phage administration, and the specificity of phages (i.e., their host range) [48]. Therefore, all these factors must be carefully evaluated in advance, for each phage treatment. Some authors state that each phage administration route holds individual challenges [45], and, thus, different forms of phage delivery have been studied and/or developed over the years with the aim to apply them in human, animal, and plant health.

### 1.2. Phage Isolation and Preparation

The success or failure of phage therapy or phage biocontrol depends, initially, on the type of phage chosen, its full characterization (physicochemical, biological, and genomic), and the preparation techniques used for producing a concentrated phage suspension. For developing phage technology aiming to serve practical purposes, it is necessary to be able to quantitate viable phage virions accurately and reproducibly [35].

For phage isolation, it is necessary to enrich an environmental sample using a target bacterial host. Such an enrichment step aims to increase the concentration of phage particles that may be present in the collected samples. After the enrichment step of water samples, a spot test is performed in a lawn of the bacterial host (the same used for the enrichment of the sample) to verify the presence of (potentially different) phage(s), by observing the formation of lysis zones indicating the death of the host bacterium [50]. In the sequence, it is necessary to determine the genomic and biological features of the phage virions, viz. the morphogenesis yield (or burst size), the latent period, the eclipse period, the intracellular accumulation period, the host range, the adsorption/desorption rates, and the titer (phage virion concentration, in plaque-forming units per mL) [39]. For effective phage action, it is still necessary to consider sufficient phage virion numbers in situ (using MOI) to achieve the adequate killing of susceptible bacterial cells [51]. Afterward, the complete purification of phages is required, especially if they are intended for human use [39]. Once isolated and duly purified, bacteriophages can be stored in liquid (suspension) or dried/lyophilized forms at 4 °C, each of which has advantages and disadvantages. The liquid form is easier to prepare and has a not-so-long average shelf half-life, but it is a form that lacks accuracy and is more prone to contamination. On the other hand, dry powders, through lyophilization or other dehydration techniques, show on average a longer preservation and stability of the phage virion’s lytic bioactivity [52].

Special attention must be given to maintaining conditions for phage viability during both storage and use. Phage virion instability is an important limitation for pharmaceutical preparations and clinical applications thereof. To avoid this, several strategies have been put forward aiming to counteract phage virion instability, with some of them relying upon (i) the modification of the viscosity of the phage medium to maintain phage virion morphology, (ii) the modification of the osmotic pressure via the addition of ions (e.g., sodium chloride) or buffer (phosphate-buffered saline (PBS)), or (iii) the use of dry phage formulations obtained via freeze drying or spray drying [34,49]. The spray drying process is used for phages in liquid phase, aiming to convert them into powdered form to integrate into formulations targeting infected wounds, bandages, pills, and inhalation delivery, among other forms [53]. Additionally, there are many biotechnological strategies available to optimize the storage of individual phages under study [34,49].

## 2. Phage Delivery Strategies for Human Health Applications

Therapy using phages in humans was utilized by Felix d’Herelle immediately after phage discovery, for the treatment of bacterial infections. In 1919, d’Herelle treated four children with dysentery, more than twenty years before the introduction of antibiotics in the 1940s [1,8,9]. Infectious diseases were the leading cause of mortality and morbidity within human populations at that time. Antibiotics, however, were very attractive due to their broad spectrum of action, allowing their use against numerous bacteria without the need to identify or characterize the bacterial pathogen, which soon led to a loss of interest in phage therapy [54].

In recent years, however, phage therapy has gained prominence due to the growing problems with multi-drug-resistant bacteria and the increase in studies that prove the success of phage therapy in the treatment of life-threatening multi-drug-resistant bacterial infections [55]. However, due to our yet incomplete understanding of phage virion biology and their interactions with bacteria inside the human body, doubts remain on whether phage therapy would be a safe treatment modality [16]. There is still no adequate knowledge on phage–phage, phage–bacteria, or phage–human interactions, mainly because of safety and efficacy concerns [56]. Nowadays, phage therapy has had a resurgence, and an increasing number of clinical trials are being conducted. Thanks to developments in genetic engineering, synthetic biology, metagenomics, high throughput sequencing, and genetic engineering, phage therapy in humans has been developed. This has prompted funding agencies to support additional investments from pharmaceutical corporations and biotech startups, as well as clinical trials [55].

Despite the success of phages in combating bacteria, proven by a myriad of research works and clinical studies, some disadvantages related to their use need to be considered, involving mainly phage virion stability and bioavailability [57]. Phage therapy has a wide range of potential applications, but it is always constrained by factors such as a limited bacterial host spectrum, immunological clearance by the human body, and the formation of anti-phage bacterial strains [44]. For the effectiveness of phages in combating bacterial infections, they must reach the sites of infection in adequate amounts (concentration) and quality (lytic viability) to bring about the eradication from the body of the bacterial pathogen responsible for the infection. Once inside the human body, the reticuloendothelial system may significantly reduce the numbers of phage virions to a low concentration, hence reducing the possibility of fighting off the pathogen [18]. Hence, there is a consensus that the future success in phage therapy depends on the effective delivery of phage therapeutics to the area of infection [45,58].

Several phage administration routes are currently being studied, together with ways to maintain phage virion lytic viability, such as oral, parenteral (intramuscular, intravenous, and intrasubcutaneous), and topical (transdermal, octic, dental, and inhalational, among other) [59,60,61].

### 2.1. Oral Administration

The oral delivery route is chosen over many other delivery methods because it offers minimally intrusive, cost-effective, and painless administration. The oral administration of bacteriophages is successful in treating gastrointestinal infections and, in certain cases, systemic illnesses. It is a route that is easily accessible and very simple to administer. However, the deactivation of bacteriophages due to the stomach’s acidic environment is the main disadvantage of the oral administration method requiring forms of phage protection [59]. Due to both the low pH prevailing in the stomach and phage virion absorption throughout the gastrointestinal tract (GIT), the outcome of phages taken orally is quite limited [62]. In a clinical study carried out using orally administered coliphages for the treatment of diarrhea in children, it was observed that the antibacterial therapy failed to accomplish the intestinal amplification of the phage virions and improve diarrhea outcomes [63]. One way to maintain phage lytic activity under the adverse conditions prevailing in the GIT is using the encapsulation of the phage virions [45].

Encapsulation may be defined as the process of the entrapment of phage particles by coating them with appropriate (bio)polysaccharides, forming a protective shell, to segregate them from the surrounding environment. Such a shell favors the protection of phage virions from external conditions and may also favor adhesion to specific susceptible bacterial cells [52]. The aim of (micro)encapsulation is, thus, to create a micro-environment in which phage virions will survive under the harsh GIT conditions, in processing and storage conditions, during desiccation processes, and other adverse biotic or abiotic conditions [64]. The advantages of the encapsulation procedure include the protection and shielding of phage virions from the outer environment. This way, it acts as an active, sustained release and delivery platform for viable phage virions over a prolonged period. The encapsulation procedure allows for the maintenance of the phage virion concentrations at therapeutically effective levels over a given time, such that once the bacterial threshold is attained, phage titers can further amplify, leading to faster and more effective antibacterial action [54].

The aggressive environment prevailing in the GIT associated with industrial (or other) processes that bacteriophages may be subject to can significantly decrease the antibacterial effect of phages [44,65]. Like other protein macromolecules, because bacteriophages are composed mainly of proteins, they are subject to the effects of protein unfolding and aggregation, as well as denaturation, resulting in the loss of lytic functionality when exposed to adverse (biotic or abiotic) conditions [66]. Hence, encapsulation techniques suitable for phage virions, aiming for targeted delivery and controlled release, are essential for future antibacterial phage therapies since phage virions need protection from environmental stresses [67]. The use of bacteriophages alone or in combination with antibacterial drugs has been recently revisited, and some studies utilizing nanosystems for bacteriophage delivery have been already reported [68]. There are many formulations for the encapsulation of phage virions in micro- and nanoparticles aiming for their effective delivery, as well as their encapsulation in stimuli-responsive systems for triggered, controlled, or sustained release at the targeted site of infection, such as reverse micelles, capsules, vesicles, core–shell particles, hybrid particle in particle (PiP) structures, emulsions, and foams [43,69].

When developing microcapsules containing encapsulated phage virions, it is necessary to preserve their lytic activity and mobility. From this perspective, research is being carried out to establish the best (bio)polymer formulations and techniques for obtaining micro- and nanoparticles. Musin et al. [70] studied the use of biodegradable polyelectrolytes, such as poly(allylamine), polyarginine, polystyrene sulfonate, dextran sulfate, and dextran, and concluded that there is a decreased viability of bacteriophages during increasing polyelectrolyte concentrations.

There is a myriad of organic and inorganic materials that can be used for preparing micro- and nanoparticles aiming to protect bacteriophage virions, among which alginate solutions associated with milk proteins such as alginate–caseinate and alginate–whey protein formulations and chitosan have been cited [71,72].

Among inorganic particles, porous calcium phosphate particles are most often used as carriers for phage virions [73]. Carbohydrate biopolymers used as matrices for microencapsulation should be non-toxic and biodegradable, capable of forming gels. Alginate and pectin are commonly used in many industrial applications [74], with alginate being one of the most commonly used organic materials for phage encapsulation due to its lack of immunogenicity, biocompatibility, biodegradability, ease of availability, and low cost [75]. Chitosan is the second most used organic polymer for bacteriophage encapsulation and delivery. Other polymer compounds used to create microcapsules are also cited in the literature, such as poly(ethylene oxide)/cellulose diacetate fibers, poly(acryl starch), and poly(lactide-co-glycolide) [73]. Synthetic polymers (such as Eudragit^®^) in association with natural polymers [76] or alone [67] have also been used for phage encapsulation. Eudragit^®^ coating imparts a greater resistance of the microparticles to the acidic environment of the stomach, and the alginate provides mucoadhesive properties to extend the residence time of the microparticles in the GIT system [77].

Encapsulation techniques, in general, are well-established procedures for several pharmaceutical drugs. Among the techniques and processes used for encapsulating microorganisms, the most common ones are spray drying, extrusion-dripping methods, emulsion, and polymerization [64]. However, many of these techniques involve the use of organic chemicals or are aggressive, which can inactivate phage virions. Hence, for phage encapsulation, the basic techniques of homogenization and extrusion have been used, which appear to be less harmful and allow the maintenance of phage lytic viability [77,78]. Techniques like emulsification, freeze drying, spray drying, encapsulation in liposomes, and electrospinning, in which bacteriophages are coated/surrounded by certain stabilizing agents, protect the virions against the external environment and have been used in phage encapsulation [66,79]. Śliwka et al. [80] developed microspheres of mannitol–alginate with the aim to obtain a dry powder phage preparation. To limit the adverse effects of the drying process, it is possible to apply different protective additives during phage encapsulation such as mannitol, trehalose, maltodextrin, sucrose, and skim milk, among others. However, it is unfeasible to propose a unique way of encapsulating phages, since the variability of phages and hosts is virtually unlimited, requiring customized approaches [57].

In the treatment of human intestinal infections, the encapsulation of lytic phages has been identified as an efficient way to protect and maintain phage lytic activity. The phages need to survive and maintain their lytic function in a highly acidic environment [66]. Table 1 outlines some research works involving encapsulated phage particles for the treatment of gastrointestinal infections in humans.

Another way to stabilize the phage particles and extend their persistence at the site of infection while allowing access to the inside of eukaryotic cells is by encapsulating the phage virions within the aqueous core of liposomes [53]. Bacteriophage virions cannot diffuse across cellular membranes, which would be a prerequisite for them to reach, infect, and lyse intracellular bacterial pathogens. Therefore, phage therapy is in general not considered to be suitable against bacteria that are inside eukaryotic cells. A promising solution to this problem lies in phage encapsulation within the aqueous core of liposomes. These lipid bilayer vesicles have good characteristics to allow their fusion with cell membranes, hence allowing cell penetration, and, thus, they may be a suitable envelope for phage virions used against intracellular bacterial pathogens [84].

Liposomes are formulated with natural phospholipids, which are biodegradable, non-immunogenic, and non-toxic. These phospholipids self-assemble and self-enclose to form spheres of lipid bilayers with an inner aqueous core which may be designed to contain hydrophilic therapeutic agents such phage virions. These advantages which are associated with their structural versatility enable the design of several liposome-based formulations [85,86]. Liposomes might also be composed of bioactive lipids, which may play a crucial role in improving the phagocytosis process of mammalian defense cells and, therefore, being efficient and non-toxic systems for carrying bioactive molecules. Liposomes are becoming a revolutionary tool in the medical–pharmaceutical field [87].

Since the development of liposomes in the 1960s, many methods for the preparation of these lipid bilayer vesicles have been developed. There are classical techniques (e.g., thin film hydration, sonication, solvent dispersion, detergent removal, and dilution) as well as sophisticated/innovative ones (e.g., use of supercritical fluids). However, whatever the method utilized, it is important to consider liposome size and size distribution (i.e., the polydispersity index), since these are crucial characteristics that affect the cargo release profile, loading capacity, biodegradation rate, and biodistribution, as well as liposome stability over the timeframe intended for the proposed goal [86].

For oral use, bacteriophages must be protected from the environmental conditions prevailing in the digestive tract, especially from the pH of the stomach. Adding to this, orally delivered phages tend to have short residence times in the GIT due to clinical symptoms such as diarrhea; this may be addressed through the mucoadhesion of liposomes with encapsulated phage virions [86]. Liposomes can prolong the persistence of liposome-encapsulated phages in the stomach and adhere to the intestinal epithelium [88]. Another interesting aspect related to the use of liposomes with encapsulated phage virions is the possibility of association with other antimicrobial compounds, such as antibiotics. The composition of liposomes and their similarity to the bacterial membrane allows their fusion with the pathogen membrane and, in this way, allows them to promote the accumulation of antibiotics within the bacteria cytoplasm, hence avoiding several mechanisms of antibiotic resistance [89]. Combating lung infections by multi-drug-resistant bacteria has also been a challenge, and liposomes have also been used for this purpose [46,87]. Table 2 displays some research works involving encapsulated phage particles within liposomes, for the treatment of human infections.

### 2.2. Topical Administration

The topical administration of bacteriophages has been reported in various situations, including treatment of superficial bacterial infections such as dermatological, burn wounds, and chronic wound/ulcer infections, which are often complicated to treat due to bacterial resistance to antibiotics [92,93], and in the treatment of deep infections arising from prosthetic joints [94,95]. Several recent studies have demonstrated the success of phage therapy in chronic wound healing.

The bacteriophage has been successfully used topically to treat skin conditions [59]. The topical route is non-invasive and has little to no first-pass effects on the phages. Generally, phage-based antibacterial treatments are less expensive than other forms of antibacterial treatment [62].

The delivery of phages, usually involving more than one type of bacteriophage (viz., a lytic cocktail), has been carried out either via pharmaceutical forms for topical use or via direct application to the skin or other body surfaces. There are several systems available for the topical delivery of phages, such as ointments, creams, gels [96,97,98], lotions, and suspensions [99,100]. Many other phage delivery systems, such as hydrogels, liposomes, nanoemulsions, adhesives, and films, have also been used for topical phage therapy [101].

For topical phage administrations, the formulation needs to be compatible with the application site, be minimally irritating to the skin, be easy to apply and remove, be stable enough to avoid the need for frequent applications, and be bacteriostatic [96,102]. In addition to these challenges, the products for the topical delivery of lytic phages need to maintain the structural integrity and viability of the phage virions, as well as adequate phage concentrations at the site of application [103].

In the development of topical preparations involving bacteriophages, several factors must be taken into account for preserving phage stability and lytic viability, with special attention to light exposure and storage temperature. Usually, phage formulations for topical applications must be stored in light-protected containers at 4 °C. Another factor of importance is the ionic nature of the semi-solid base. The overall electrostatic charge of phages (i.e., their Zeta potential) changes with the pH of the environment, and phage capsids and tails can carry opposite charges [104].

A pharmaceutical form for topical application widely used for phage delivery is hydrogel. Hydrogels are an excellent alternative for dermal asset administration because they are malleable, flexible, and biocompatible, minimizing the irritation of natural tissues while allowing the incorporation of substances that enhance skin permeation [105,106]. The three-dimensional network architecture of hydrogels is due to the presence of crosslinks between the (bio)polymeric chains. Due to these cross-links, hydrogels tend to exhibit elastic and sometimes viscoelastic behavior. They can be formulated from a wide variety of either natural or synthetic polymers. Crosslinking between polymer chains can be provided by hydrogen bonding, van der Waals interactions, and covalent bonds [107]. Hydrogels are an interesting delivery vehicle for bacteriophages as they keep the wound moist, act as a protective barrier, and facilitate the wound healing process, being widely used for the delivery of bacteriophages in association (or not) with different active compounds to fight infections in the skin [108]. Neutral hydrogels must be applied for carrying phage preparations, aiming for the stability of the phage virions, since products with high acidity exert severe adverse effects on the phage virions. In addition, phage stability/activity can also be affected by active compounds and adjuvants such as anti-infective substances [109,110]. In Table 3, one can see some examples of hydrogel formulations aiming to carry bacteriophages for the treatment of skin infections.

### 2.3. Parenteral Administration

Many researchers demonstrated that parenteral phage therapy can be safe and effective under certain circumstances, and intravenous bacteriophage therapy was well tolerated without significant side effects [62,116]. The parenteral method of administration is the most beneficial in terms of therapeutic benefit because it offers immediate distribution and maximum uptake. Nevertheless, there has been little data available on the pharmacokinetic profiling of phage formulations [59]. In addition, all phage preparations used for this route of administration need to be extensively purified before use [62].

Many clinical situations can potentially benefit from the intravenous administration of phages, including cystic fibrosis and lung transplants, bone and prosthetic joint infections, ventricular assist device infections, recurrent urinary tract infections, and bacteremia, among others [117,118,119]. Table 4 displays several examples of human infections treated with phages administered intravenously. It has also been noticed that most of the time, infections treated with phages via the intravenous route are associated with the systemic administration of antibiotics [118].

The biggest problem that has been verified with the parenteral route for the administration of phages is the possibility of quick phage elimination by the immune system when these antibacterial entities are administered intravenously [13,120]. However, according to Speck and Smithyman [117], a rapid removal of injected phages from the bloodstream occurs when there is a complete lack of host bacteria in which they could replicate.

**Table 4:** Examples of human infections treated with phages administered intravenously.

Type of Infection	Pathogen(s)	Treatment	Reference
Prosthetic joint infections	Methicillin-resistant *Staphylococcus aureus* (MRSA)	Intra-articular and intravenous bacteriophage therapy	[121]
Respiratory infection	Multi-drug-resistant (MDR) *Acinetobacter baumannii*	Intravenous and nebulized bacteriophage therapy with systemic administration of antibiotics	[122]
Respiratory infection (cystic fibrosis awaiting lung transplant)	Multi-drug-resistant (MDR) *Pseudomonas aeruginosa*	Intravenous bacteriophage therapy (IVBT) with systemic administration of antibiotics	[123]
Prosthetic joint infection	Methicillin-resistant *Staphylococcus aureus* (MRSA)	Intraoperative and intravenous bacteriophage therapy	[124]
Prosthetic joint infection	Persistent methicillin-sensitive *Staphylococcus aureus* (MSSA)	Intravenous and intra-articular infusions of bacteriophage therapy	[125]
Refractory ventricular assist device infection (heart infection)	Multi-drug-resistant (MDR) *Pseudomonas aeruginosa*	Intravenous phage therapy with systemic administration of antibiotics	[126]
Bone infection	Multi-drug-resistant (MDR) *Pseudomonas aeruginosa*	Locally administered phages and intravenously administered phages with systemic administration of antibiotics	[127]
Necrotizing pancreatitis	Multi-drug-resistant (MDR) *Acinetobacter baumannii*	Intravenous and intracavitary administration of phages	[128]

### 2.4. Airways Administration

The current practice for treating human respiratory disorders involves inhaling medications through the mouth and into the airways. Inhaled medications bypass the significant nose cavity barriers by entering through the mouth cavity, which results in larger amounts reaching the lungs [62]. The most recent advances in the field of phage therapy have been inhalation technologies. Phage application is rapid and allows the delivery of phages directly into the airways. However, it requires the use of appropriate inhalers [59].

Bacteriophages can be administered intranasally, as an aerosol or nasal spray, to treat bacterial infections of the respiratory tract. The first reports of successful inhalation therapy for treating lung infections using phages appeared around 1960, with some studies of phage inhalation therapy performed in humans and animals [129,130]. At the moment, pulmonary phage therapy has been predominantly applied in people who suffer from muco-obstructive lung diseases such as cystic fibrosis, non-cystic fibrosis, bronchiectasis, primary ciliary dyskinesia, and chronic obstructive pulmonary disease [131]. Besides this, a small number of phages have been isolated and utilized to fight common bacterial infections in the lungs, due to *Streptococcus pneumoniae*, *Haemophilus influenzae*, *Staphylococcus aureus*, *Mycobacterium tuberculosis*, *Corynebacterium*, *Neisseria*, *Fusobacterium*, *Moraxella catarrhalis*, and *Pseudomonas aeruginosa* [52].

The success of phage therapy via airways requires, first, the selection of both the most suitable phages and the most appropriate inhalation device based on empirical in vitro assessments. Studies performed in in vitro models to evaluate phage inhalation showed a reduction in phage concentration (i.e., phage titer) associated primarily to the process of nebulization [132]. Another challenge is developing an adequate phage formulation for the inhalational administration via nebulization [133], with the size of particles containing the phage virions needing to be such that they reach, for example, the alveoli, where bacterial colonization occurs [62]. The association with antibiotics is also routine for the success of phage inhalation therapy [134]. The results obtained in the several research works found in the specialty literature demonstrate that bacteriophages can be administered safely via inhalation or respiratory routes. Table 5 displays several examples of human infections in the upper or lower respiratory tract treated with phages administered via airway routes.

In general, it can be said that little is known about optimal phage treatment durations or routes of phage administration to use in the treatment of different bacterial infections, and clinical trials are needed to determine the adequate duration of bacteriophage therapy [121]. Usually, treatments with phages have been carried out considering each patient case individually. Hence, future research is required to determine if lytic phages should be utilized either independently or as a lytic cocktail, or even in conjunction with antibiotics. Antibacterial combination therapy has the potential to be used in clinical settings to effectively treat patients and avoid or minimize the development of bacterial resistance, since phages and antibiotics may display a synergistic effect as suggested by several researchers [61].

However, the use of bacteriophages in human health to fight bacterial infections where antibiotics no longer work still faces serious challenges. Bacteriophages and their byproducts (e.g., enzymes such as lysins) should undergo the same process as chemical drugs for them to be able to receive regulatory authorization for use and manufacturing for commercialization. To prove the effectiveness, safety, and stability of bacteriophages and their enzymes, clinicians and researchers must set up large-scale clinical trials. However, the lack of clear guidelines and regulations has made bacteriophages less desirable to pharmaceutical companies and funding agencies, making it difficult for both clinicians and researchers to set up these trials [139]. The development of stable pharmaceutical formulations integrating phage virions, taking into account aspects related to controlled release and targeted delivery to meet the needs of infectious disease management, is of utmost importance for a future “phage industry”. In addition, such research must also support scalable, cost-effective manufacturing processes for the production of phage-based products [140].

## 3. Phage Delivery Strategies for Animal Health and Food Applications

One of the first reports of the use of phages in veterinary medicine was against *Salmonella gallinarum* strains derived from birds in France in 1919 by Felix D’Herelle. After this first use, another use of bacteriophages in veterinary medicine was described by Pyle, in 1926, to treat Salmonellosis in chickens (caused by *Salmonella enterica* serotype *pullorum*), but the results were not good. At that time, it was unknown that certain bacteriophages may be rendered inactive by either the digestive tract enzymes or the gastric acidity [141]. However, the first experimental phage therapy trial in veterinary medicine was performed in 1941, where the researchers first isolated phages against *Staphylococci* from cow milk and then evaluated their effectiveness against mastitis caused by *Staphylococcus* sp. [142]. Sadly, research on the therapeutic potential of bacteriophages in phage therapy was neglected and eventually faded away with the advent and large-scale production of antibiotics [141]. Nevertheless, the quantity and quality of research papers on bacteriophage therapy with possible veterinary applications have significantly increased over the last two decades [143].

**Figure 4:**
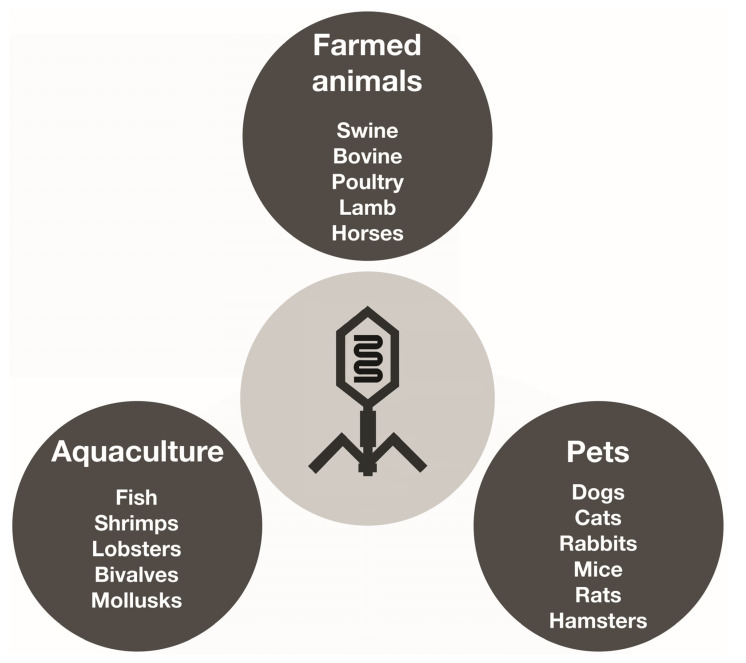
Different animal species that can be treated with phagotherapy in farms, aquaculture settings, and also types of companion animals.

However, the excessive use and misuse of antibiotics has led to the emergence of multidrug-resistant bacteria, and this has rekindled interest in the creation of alternatives to antibiotics, such as using bacteriophages to manage bacterial infections in veterinary medicine settings, just like in human medicine [144,145,146]. Currently, bacteriophages have been employed to control a variety of bacterial infections caused by *Salmonella*, *E*. *coli*, *Listeria monocytogenes*, *Campylobacter*, *Cronobacter sakazakii*, *Staphylococcus aureus*, and *Shigella* in several animal species [25,147]. Figure 4 displays different animal species that can be treated with phagotherapy in farmed animals, both in land and aquatic systems, and also types of companion animals. Phage therapy has already been successfully used to treat infections by *E*. *coli* in pigs, lambs, and calves [25]. Bacteriophages can be used in a variety of forms and methods to control and eliminate bacteria, including therapy, food protection, and sanitation procedures. The effectiveness of phage therapy in animal health has been significant [145]. Phagotherapy was used to treat calves, lambs, and pigs infected with *E*. *coli*. Phages are also successfully used against skin infections caused by *P*. *aeruginosa* in animals [25]. Table 6 shows some other examples.

In addition to the various therapeutic uses of bacteriophages, these antibacterial entities can be employed also at all levels of food production [18]. Phages can be used in the food industry at many stages of the production line: (i) directly, on both plants and animals, to reduce the risk of bacterial contamination, infection, and disease; (ii) in food processing facilities to avoid the formation of bacterial biofilms [157]; or (iii) directly on foodstuff, to preserve the product from bacterial contamination [36,158]. Foodborne diseases are one of the main causes of morbidity and mortality worldwide and are considered one of the biggest public health problems. In addition, the economic impact of foodborne bacterial infections is considerable [41]. Although conventional antibacterial techniques such as pasteurization, high-pressure processing, irradiation, and chemical disinfection can reduce bacterial populations in foodstuff to varying degrees, these methods also have significant drawbacks such as high initial investment, the possibility of processing equipment damage due to their corrosive nature, and a negative impact on the organoleptic properties (and possibly on the nutritional value) of foods. In this way, bacteriophage-based biocontrol is a potentially viable method that avoids many such drawbacks [159]. However, according to Kazi and Annapure [147], “the recent approval of bacteriophages as food additives has opened the discussion about edible viruses”. To fully comprehend the processes by which hosts develop phage resistance and the strategies for surmounting it, more research is still required for applying phages to food as a means of eliminating or preserving contamination by pathogenic bacteria. Nowadays, there is extensive research being carried out on the efficacy of using phages as antibacterial agents, specifically in food animal production, aiming to control bacterial diseases [22]. Currently, there are already products containing phages that were approved with GRAS status (Generally Recognized as Safe) by the U.S. Food and Drug Administration (FDA) for use in bacterial pathogen control in food processing, including ready-to-eat foods and poultry products [160]. For example, commercially available ListexP100™ (Micreos Pharmaceuticals AG, Zug, Switzerland) and ListShield™ (Intralytix Inc., Columbia, MD, USA) solutions are recommended for lowering the levels of *Listeria monocytogenes* on non-food equipment as they help to avoid the formation of biofilms and may even aid in the eradication of this pathogen [161].

As in human therapy, the route of phage administration, phage dose, and form of phage delivery are important parameters to be taken into consideration. Phages can be administered to animals mainly through oral and topical routes [162].

### 3.1. Oral Administration

Usually, phages for phage therapy in animals are administered orally, either as a feed supplement, in water or using a gavage. However, in intensive animal farming, gavage administration is not suitable for obvious reasons. Due to the poor stability of phages in acidic environments like the stomach, the oral administration of phages poses limits in terms of phage delivery techniques. Many publications have discussed this issue and suggested the administration of phages with buffering chemicals [162].

Keerqin et al. [163] used bacteriophages in a buffer solution for administration to chickens. Those researchers evaluated the action of bacteriophages (as a lytic cocktail) in the treatment of chickens against *Clostridium perfringens*-induced necrotic enteritis. The phage cocktail was prepared in phage buffer and administered via gavage. The experimental results of the study by Keerqin et al. [163] indicated that bacteriophage treatment was promising for protecting the intestinal health of poultry from *Clostridium perfringens*-induced necrotic enteritis.

In another research work aiming to combat Salmonellosis in broilers, a phage cocktail prepared in magnesium salt buffer was administered via gavage to broilers raised in commercial conditions. The contamination by Salmonella was significantly reduced from over 70% down to 0% prevalence after four days of phage treatment [164]. Phage efficacy was also verified in the treatment of chickens inoculated with *Salmonella enterica* serovar Typhimurium using phages suspended in a saline solution. The efficacy of the phage therapy was similar when compared to treatment using the antibiotics enrofloxacin and colistin [165].

Clavijo et al. [166] evaluated the effectiveness of a commercial bacteriophage cocktail named SalmoFREE^®^ (cocktail with 6 *Salmonella* lytic bacteriophages, produced by SciPhage (Bogotá, Colombia)) administered in the drinking water in a commercial broiler farm to control *Salmonella*, and the results obtained revealed that the phage cocktail controlled the incidence of *Salmonella* and did not affect the animals or the production parameters in any way whatsoever.

On the other hand, phage encapsulation is an alternative that allows the stable and controlled delivery of bacteriophages. Encapsulated phage virions are protected from the harsh environment of the stomach, with the carrier particles facilitating phage retention during passage through the GIT to ensure a successful therapeutic effect. Several research studies have indicated that encapsulation can increase phage lytic viability in harsh acidic pH values (between 2 and 3) and allows phage virions to better withstand higher temperatures (up to 60 °C) than their free counterparts [149,150]. Furthermore, Soto et al. [149] observed that phages encapsulated in a calcium alginate matrix had their presence extended for an extra 100 h in a water flow system, which simulated the automatic birdbaths used in the poultry industry, when compared to non-encapsulated phages. In another research work [23], the development of calcium alginate microparticles involving a mixture of gelatin, calcium chloride and chitosan, to encapsulate a cocktail of two lytic phages against *Salmonella enterica* has been reported, aiming at the oral treatment of Salmonellosis in chickens. Table 6 shows different experiments using encapsulated phages for animal treatments.

Using phages for the treatment of infections in aquaculture can also be performed through the delivery of phages via food, in baths, or in the environment. Phage therapy in disease control management in aquaculture has proven its efficacy, although many challenges still exist. Phage therapy in aquaculture became a greener and safer alternative to the use of antibiotics [167]. Phage-based pathogen targeting has a variety of uses in both fish and the environment where they are raised [168].

The disease-causing bacteria in fish are frequently spread via water bodies in aquaculture settings. The lack of effective vaccines against various bacterial diseases that harm fish in the aquaculture industry makes it quite difficult to avoid infectious diseases in fish [168]. Typically occurring bacterial infections in aquaculture that cause production output failure or decline are primarily associated to Gram-negative bacteria such as *Aeromonas hydrophila*, *A*. *salmonicida*, *Edwardsiella tarda*, *Flavobacterium psychrophilum*, *Pseudomonas fluorescens*, and various *Vibrio* species. On the other hand, diseases caused by Gram-positive bacteria such as *Streptococcus iniae*, *Renibacterium salmoninarum*, or *Mycobacterium* spp. are much less frequent [169].

The first application of phage therapy for bacterial control in fish dates back 1981, in Japanese eel *Anguilla japonica* [167]. After this first phage therapy experiment, many others were carried out and several lytic bacteriophages have been isolated to various pathogenic bacteria, viz. *Edwardsiella tarda*, *Edwardsiella ictaluri*, *Lactococcus garvieae*, *Pseudomonas plecoglossicida*, *Streptococcus iniae*, *Flavobacterium columnare*, *Flavobacterium psychrophilum*, *Aeromonas salmonicida*, *Aeromonas hydrophila*, *Vibrio anguillarum*, *Vibrio harveyi*, and *Vibrio parahaemolyticus* [170].

Kunttu et al. [168] evaluated the use of phages to prevent and treat infections by *Flavobacterium columnare* in rainbow trout by using several phage delivery techniques such as via baths, via phage-coated materials, and via oral delivery in fish feed. In this case, the most effective phage therapy procedure consisted in applying phages via bath exposure immediately after the first symptoms of the disease appeared in the fish population.

Due to the advantages of phage therapies, there has been an increasing number of research studies on the biology and biotechnology of bacteriophages aiming at finding novel and eco-friendly ways to control pathogenic bacteria in aquaculture.

### 3.2. Topical Administration

The topical administration of phages in animals has been extensively studied due to the ease of application, particularly in companion animals. One of the first antibacterial treatments using phages was for the treatment of otitis in dogs and was described by Hawkins and colleagues [171]. A group of 10 dogs received, directly into the auditory canal of one ear, a single dose of a topical preparation containing a cocktail of six lytic bacteriophages against *P*. *aeruginosa*. Forty-eight hours after the beginning of the treatment, the clinical score and *P*. *aeruginosa* counts in all animal’s ears had fallen.

Transdermal permeation can also be used for phage delivery in animals. Silva et al. [172] developed a hydroxyethylcellulose gel containing the ionic liquid choline geranate for the transdermal delivery of lytic bacteriophages against *Staphylococcus intermedius*, aiming to treat pyoderma in animals.

The subcutaneous route can also be an alternative for phage delivery. In a study conducted by Solomon et al. [173], the subcutaneous route was used for the delivery of phages in association with systemic antibiotic therapy in dogs. In this study, the dogs received shots of 0.5–1.0 mL of StaPhage^®^ Lysate (SPL) subcutaneously once or twice a week for 20 weeks. An effectiveness of more than 80% relative to the control of pruritus along with regression of the lesions was observed.

The use of vaginal eggs and suppositories has also been reported for phage delivery in the treatment of animal infections. Balcão and collaborators [174] developed a vaginal egg formulation for the delivery of lytic phages against *E*. *coli* to fight pyometra infections in female cats and dogs. In another research work found in the specialty literature, the treatment of diarrhea in young calves was carried out using suppositories containing probiotic strains of *Lactobacillus* spp. and lytic bacteriophages for pathogenic *E*. *coli* [146]. The suppositories led to a reduction in the duration of diarrhea, completely eliminating it within 24–48 h after the beginning of treatment.

Phage therapy modality varies depending on the complexity, location, and target bacteria, which is shown in recent research on intensively farmed animals. However, the use of phage therapy has demonstrated that bacterial pathogens causing infections in animals can be significantly decreased or even eliminated by utilizing phages [27].

Nevertheless, despite the effectiveness and safety of phage therapy in animals, the use of phages is still not specifically regulated by law [141].

## 4. Phage Delivery Strategies for Plant Health

Some bacterial infections in plants can cause illness, which can result in significant financial losses in agriculture by lowering both production yields and product quality. In this regard, phages may be a viable solution in agriculture for minimizing plant infections and their harmful effects [175,176].

One of the first studies using bacteriophages for phytopathogen bacteria biocontrol in plants dates back to 1926. Phages were used to fight *Erwinia carotovora* subsp. *atroseptica* (now called *Pectobacterium carotova* subsp. *Atroseptica*), responsible for potato tuber rot [177]. However, nowadays, the indiscriminate use of antibiotics, chemicals, and copper-based formulations as antibacterial treatments for crops has led to the development of resistant phytopathogen bacterial strains, and, in addition, the continuous use of these substances is highly harmful to the environment [177,178,179].

Several plant bacterial pathogens have been combated using phage therapy. The most relevant phytopathogen bacteria responsible for damaging crops and affecting food production around the world are *Pseudomonas* spp., *Xanthomonas* spp., *Pectobacterium* spp., *Ralstonia* spp., *Burkholderia* spp., *Dickeya* spp., *Clavibacter ichiganensis*, and *Agrobacterium tumefaciens* [177]. To combat these phytopathogens, the bulk of isolated phages are tailed ones (mainly from the *Podoviridae* or *Myoviridae* families), although the use of filamentous phages has also shown considerable potential [32].

There is a lot of research being conducted involving phages to combat phytopathogen bacteria in various plant species and, obviously, depending on the characteristics of plantations and bacterial diseases, different phage delivery strategies are under scrutiny. Because phages exclusively target certain bacterial hosts, they are ideal for agricultural purposes since the protective bacterial flora of plants is not affected by the use of lytic phages [176,180]. Table 7 shows the use of different phages for plant crop treatments.

Phages can be applied in field conditions, including immersion (plant seeds or plant seedlings are immersed in a phage cocktail solution before planting), infiltration (phages are directly injected into plant tissues (xylem) using an injection gun), spraying (bacteriophages are applied topically to the phyllosphere), and soil drenching (bacteriophages are applied to the rhizosphere) [179]. The majority of lytic phages against plant phytopathogen bacteria, however, show greater sensitivity to abiotic variables such as UV radiation incidence in the plant’s phyllosphere, bacterial habits, and rhizosphere habitats [195]. In addition, the effective mitigation of bacterial contamination requires that high phage numbers are present close to the phytopathogen at critical times in the disease cycle [196]. Nevertheless, the biggest problems related to the use of bacteriophages in combatting bacterial infections in plantations lie in the excessive specificity of bacteriophages, adding to the low resistance of viral particles to extreme abiotic conditions [185]. Hence, strategies can be proposed for phage virion protection, such as the best time of day for phage application (typically at dusk), the use of stabilizing agents, and daily or weekly applications, among other strategies [197].

Aiming to enhance the effectiveness of bacteriophage treatment for controlling bacterial spot in plants, Balog et al. [198] developed three formulations for phage protection. The formulations developed by those researchers, combining sucrose, Casecrete NH-400 (a water-soluble casein protein polymer), pregelatinized maize flour, and/or skim milk, boosted the effectiveness of phages against *Xanthomonas campestris* pv. *vesicatoria* in both greenhouse and field testing. In another survey by Ibrahim et al. [181], a bacteriophage formulation integrating skim milk and sucrose in association with acibenzolar-S-methyl (ASM, a fungicide) was applied against *Xanthomonas citri* subsp. *citri*. The application of the phage formulation was carried out on leaves of Mexican lime (*Citrus aurantifolia*) in both greenhouse and field conditions. The results obtained with this phage formulation in association with ASM were superior to those obtained with copper-based bactericides [181]. In yet another study, better results were also observed when bacteriophage virions were associated with ASM in the treatment of bacterial infections by *Xanthomonas axonopodis* pv. allii, a phytopathogen responsible for the onion leaf blight [199].

Gašić et al. [200], using also a formulation with skim milk and sucrose, managed to extend phage survival in ex vivo assays. The phages survived for at least 7 d on the surface of pepper plant leaves in greenhouse conditions, showing the ability to persist on plant tissues without the presence of the host bacterium. A formulation with skim milk was also used with phages to fight *Anthomonas axonopodis* pv. *punicae*, a phytopathogen responsible for infections in pomegranate plants. The phage mixture reduced the severity of bacterial blight when the plants were treated with formulated phages in skim milk, simultaneously enhancing plant growth [201].

Other substances can also be associated with phages. In another study, phage PE204 was used as a model lytic bacteriophage to investigate its biocontrol potential for bacterial wilt on tomato plants caused by *Ralstonia solanacearum*. The findings suggested that an application of phage with a surfactant via drenching the soil around the plant may be used to reduce bacterial contamination of cultures [182].

Bacteriophages may be useful in the biological control of bacterial plant diseases, according to published data, but there are still significant challenges that must be overcome. Whatever the agricultural application of phages, the process itself needs to be more clearly written out, organized, and defined by laws. Phage development systems for agriculture and food have their distinct processes, protocols, and challenges such as the following: (i) specific phage selection and discovery; (ii) therapeutic proof of concept; (iii) technology development; (iv) market identification; (v) technology transfer; (vi) commercial scale-up; (vii) regulatory/registration procedures; and (viii) end-user technology adaptation [202].

Generally, phage therapy and phage biocontrol have several advantages over antibiotics, besides being an environmentally friendly method of treating dangerous bacterial infections, even when antibiotics are ineffective. However, despite the countless advantages of phage-based biocontrol, there are still difficulties and issues to be addressed and solved, including (but not limited to) the following: (i) the host range of phages may be too narrow, as while narrow target specificity has benefits, serotype specificity may restrict phage effectiveness; (ii) the need for protection from harsh environmental conditions; (iii) the safe propagation of phages specific for phytopathogenic strains; (iv) the standardized purification of phages suitable for bacterial biocontrol applications; and (v) the specific rules and laws addressing phage utilizations [203].

## 5. Conclusions

Despite numerous successful attempts to use bacteriophages to combat bacterial infections, there are still many hurdles to overcome. This is mainly due to the lack of a specific regulatory framework that meets the requirements of a flexible and personalized treatment such as the phagotherapy model for large-scale demand. One of the main challenges in regulating phage use approval is the diversity of phages that will be required to successfully implement an antibacterial therapeutic strategy. Furthermore, to harness the full potential of phage therapy, there should be room to make changes to phage-based formulations without the need for a lengthy and expensive approval process by government agencies. The standardization of in vivo assays that provide an evaluation of the efficacy and safety of a standardized phage product is still necessary. Finally, for phagotherapy in humans and animals, more and larger preclinical and clinical trials are needed to confirm the effectiveness of this type of antibacterial therapy. The phage therapy of human and animal infections and the phage-based biocontrol of phytopathogenic bacteria harness the power of nature’s tiny bacterial predators and are a ray of light amidst the increasing darkness that engulfs the blue sphere that we call home.

## Figures and Tables

**Table 1:** Encapsulated phage formulations for the delivery of bacteriophage particles aiming to treat gastrointestinal infections in humans.

Pathogen(s)	Microparticle Production Technique and Composition	Reference
*Salmonella enterica*, *Shigella flexneri* and *Escherichia coli*	Sodium tripolyphosphate; gelation; acetic acid and chitosan	[72]
*Escherichia coli*	Sodium alginate and low methoxylated (LM) pectin (33% esterification degree)	[74]
*Escherichia coli*	Sodium alginate and chitosan–acetate buffer solution	[81]
*Escherichia coli*	Sodium alginate and chitosan, polyethylene imine (PEI)	[82]
*Proteus vulgaris* and *Escherichia coli*	Na_2_CO_3_ and CaCl_2_ (microspherolits) with alternate adsorption of oppositely charged polyelectrolytes (polyelectrolyte microcapsules)	[73]
*Escherichia coli*	Chitosan–alginate coating shell	[83]

**Table 2:** Liposomes containing encapsulated phage virions for the treatment of human infections.

Bacterial Infection(s)	Pathogen(s)	Liposome System Composition	Reference
Gastrointestinal infection	*Escherichia coli*	Low crosslinked anionic nanocellulose-based hydrogels (prepared using 2,2,6,6-tetramethylpiperidine-1-oxyl (TEMPO)-oxidized cellulose nanofibers (TOCNF)) with entrapped phages, crosslinked within highly porous spherical polyHIPE particles	[90]
Gastrointestinal, spleen liver and muscle infections	*Salmonella enterica* serovar *Typhimurium*	1,2-dilauroyl-rac-glycero3-phosphocholine (DLPC), cholesteryl polyethylene glycol 600 sebacate (Chol-PEG600), cholesterol (Chol), and cholesteryl 3β-N (dimethylaminoethyl) carbamate hydrochloride (cholesteryl) in a molar ratio of 1:0.1:0.2:0.7	[88]
Pulmonary infection	*Staphylococcus pneumoniae*	1,2-dioleoyl-3-trimethylammonium propane chloride (DOTAP) and 1,2-dioleoyl-sn-glycerophosphocholine (DOPC) (30:70) and levofloxacin (1:100, levofloxacin to lipids)	[89]
Gastrointestinal infection	*Escherichia coli* and *Staphylococcus aureus*	Phospholipid DSPC (1,2-distearoyl-sn-glycero-3-phosphocholine) and cholesterol (1:1)	[86]
Blood infection	*Klebsiella pneumoniae*	Phosphatidylcholine:Cholesterol:Tween 80:Stearylamine (9:1:2:0.5)	[47]
Skin infection	*Staphylococcus aureus*	Phosphatidylcholine:Cholesterol:Tween 80:Stearylamine (7:3:1:0.5) and phages (MR-5 and MR-10)	[85]
Skin burn wound infection	*Klebsiella pneumoniae*	Phosphatidyl choline:Cholesterol:Tween-80: Stearylamine (8:2:1:0.5)	[91]
Pulmonary infection	*Klebsiella pneumoniae*	Phosphatidylcholine:Cholesterol:Tween 80:Stearylamine (9:1:2:0.5)	[46]
Pulmonary infection	*Pseudomonas aeruginosa*	L-α-phosphatidic acid (0.05 mg/mL) and silicone (0.05 mg/mL)	[87]

**Table 3:** Examples of hydrogels as carriers of bacteriophages for the treatment of skin infections.

Pathogen(s)	Hydrogel System Composition	Reference
*Pseudomonas aeruginosa*	Blends of hydroxyethyl cellulose (HEC), hydroxypropyl methylcellulose (HPMC), polyethylene oxide (PEO), polyvinyl alcohol (PVA), hydroxypropyl cellulose (HPC) and polyvinylpyrrolidone (PVP), and phages	[108]
*Staphylococcus aureus*	Blends of polyvinyl alcohol (PVA) and sodium alginate (SA) crosslinked by boric acid and calcium ions, and MR10 phage together with minocycline	[111]
Staphylococci, Streptococci, Enterococci, *Proteus* spp. (*P*. *vulgaris*, *P*. *mirabilis*), *Klebsiella* spp. (*K*. *pneumoniae*, *K*. *oxytoca*), *Pseudomonas aeruginosa* and *Escherichia coli*	Polyvinyl alcohol (PVA), aqueous solution of bacteriophages “*Complex Pyobacteriophage*” and succinic acid; Polyvinyl alcohol (PVA) and aqueous solution of bacteriophages “*Complex Pyobacteriophage*” and lidocaine; Polyvinyl alcohol (PVA) and aqueous solution of bacteriophages “*Complex Pyobacteriophage*” and succinic acid and lidocaine	[103]
*Escherichia coli*	Polyvinyl alcohol (PVA) hydrogel and bacteriophage	[112]
*Acinetobacter baumannii*	Sodium alginate, choline oleate and lytic bacteriophages	[105]
*Escherichia coli*	Sodium alginate, gelatin, and hyaluronic acid for encapsulating acid fibroblast growth factor (aFGF) and bacteriophages	[113]
*Pseudomonas aeruginosa*	Polycaprolactone (PCL) nanofibers (non-woven textile) and bacteriophages	[110]
*Staphylococcus aureus*	Beta-glucan (BG), arabinogalactan, gums, and bacteriophages	[114]
*Enterococcus faecalis*	Alginate-nanohydroxyapatite hydrogel and phages	[115]

**Table 5:** Examples of human infections treated with phages administered via airway routes.

Type of Infection	Pathogen(s)	Treatment	Reference
Pneumonia	Methicillin-resistant *Staphylococcus aureus* (MRSA)	Association with antibiotics	[134]
Pneumonia	*Staphylococcus aureus*	Inhalation and intravenous injection	[135]
Pulmonary infection and severe cystic fibrosis lung disease	*Mycobacterium abscessus*	Inhalation	[136]
Pulmonary infection (double lung transplant due to cystic fibrosis)	*Achromobacter xylosoxidans* pan-drug-resistant (PDR)	Nebulization	[137]
Cystic fibrosis	*Pseudomonas aeruginosa*	Nasal spray and inhalation solutions	[138]

**Table 6:** Encapsulated phage formulations for the delivery of bacteriophage particles aiming to treat bacterial infections in animals.

Pathogen(s)	Microparticle Production Technique and Composition	Animal	Reference
*Salmonella T* *yphimurium*	Sodium alginate/CaCO_3_	Broiler chickens	[148]
*Salmonella enteritidis*	Sodium alginate/CaCO_3_	Broiler chickens	[149]
*Salmonella enterica*	Gelatin/CaCl_2_/chitosan	Poultry	[23]
*Eschericha coli*	Sodium alginate/CaCO_3_	Broiler chickens	[150]
*Colibacillosis*	Chitosan	Broiler chickens	[151]
*Salmonella enteritidis*	Sodium alginate/CaCl_2_	Chickens	[152]
*Salmonella* Senftenberg	Eudragit™ polymer L100/trehalose	Poultry	[153]
*Salmonella enteritidis*	Xanthan gum/sodium alginate/CaCl_2_/chitooligosaccharide	Chickens	[154]
*Salmonella typhimurium*	Alginate/whey protein	Broiler chickens	[155]
*Pseudomonas aeruginosa*	Dual antibiotic–phage delivery system containing hydrogel and alginate microbeads loaded with a phage cocktail plus meropenem	Mouse	[156]

**Table 7:** Examples of the use of different phages for plant crop bacterial infection treatments.

Phytopathogen(s)	Plant Species	Application	Reference
*Xanthomonas citri* subsp. *citri*	Mexican lime	Phyllosphere	[181]
*Ralstonia solanacearum*	Tomato plants	Rhizosphere	[182]
*Ralstonia solanacearum*	Potato plants	Phage injection into the plant’s xylem and soil drenching	[183]
*Ralstonia solanacearum*, *Pectobacterium carotovorum* and *Pectobacterium atrosepticum*	Potato plants	Soil drenching	[184]
*Curtobacterium flaccumfaciens*	Soybean	Leafs, by airbrushing	[185]
*Erwinia amylovora and Erwinia pyrifoliae*	Apple plants	Leafs, by compression spray	[186]
*Xanthomonas euvesicatoria* pv. *perforans*	Tomato plants	Phyllosphere	[187]
*Pseudomonas syringae* pv. *tomato*	Tomato plants	Soil drenching and phyllosphere spraying	[188]
*Dickeya solani*	Potato plants	Tubers	[189]
*Xanthomonas axonopodis* pv. *punicae*	Pomegranate plants	Phyllosphere	[190]
*Xanthomonas oryzae*	Rice	Rice seeds	[191]
*Pseudomonas syringae* pv. *actinidiae*	Kiwifruit plants	Phyllosphere	[192]
*Pseudomonas syringae* pv. *actinidiae*	Kiwifruit plants	Excised leafs	[193]
*Pseudomonas syringae* pv. *garcae*	Coffee plants	Excised leafs	[194]

## Data Availability

The data presented in this study are available in this article.

## References

[1] Lin D.M., Koskella B., Lin H.C. (2017). Phage therapy: An alternative to antibiotics in the age of multi-drug resistance. World J. Gastrointest. Pharmacol. Ther..

[2] Gamachu S.B., Debalo M. (2022). Review of bacteriophage and its applications. Int. J. Vet. Sci. Res..

[3] Harada L.K., Silva E.C., Campos W.F., Del Fiol F.S., Vila M., Dąbrowska K., Krylov V.N., Balcão V.M. (2018). Biotechnological applications of bacteriophages: State of the art. Microbiol. Res..

[4] Dublanchet A., Bourne S. (2007). The epic of phage therapy. Can. J. Infect. Dis. Med. Microbiol..

[5] Zhang M., Zhang T., Yu M., Chen Y.L., Jin M. (2022). The life cycle transitions of temperate phages: Regulating factors and potential ecological implications. Viruses.

[6] Kakasis A., Panitsa G. (2019). Bacteriophage therapy as an alternative treatment for human infections. A comprehensive review. Int. J. Antimicrob. Agents.

[7] Wernicki A., Nowaczek A., Urban-Chmiel R. (2017). Bacteriophage therapy to combat bacterial infections in poultry. Virol. J..

[8] Diallo K., Dublanchet A. (2023). A century of clinical use of phages: A literature review. Antibiotics.

[9] Clark J.R. (2015). Bacteriophage therapy: History and future prospects. Future Virol..

[10] Wdowiak M., Paczesny J., Raza S. (2022). Enhancing the stability of bacteriophages using physical, chemical, and nano-based approaches: A review. Pharmaceutics.

[11] Żbikowska K., Michalczuk M., Dolka B. (2020). The use of bacteriophages in the poultry industry. Animals.

[12] De Angelis L.H., Ponsecchi G., Fraziano M., D’Andrea M.M. (2022). Application of bacteriophages for human health: An old approach against contemporary “bad bugs”. Microorganisms.

[13] Chen Q., Dharmaraj T., Cai P.C., Burgener E.B., Haddock N.L., Spakowitz A.J., Bollyky P.L. (2022). Bacteriophage and bacterial susceptibility, resistance, and tolerance to antibiotics. Pharmaceutics.

[14] Cisek A.A., Dąbrowska I., Gregorczyk K.P., Wyżewski Z. (2017). Phage therapy in bacterial infections treatment: One hundred years after the discovery of bacteriophages. Curr. Microbiol..

[15] Domingo-Calap P., Delgado-Martínez J. (2018). Bacteriophages: Protagonists of a post-antibiotic era. Antibiotics.

[16] Lawrence D., Baldridge M.T., Handley S.A. (2019). Phages and human health: More than idle hitchhikers. Viruses.

[17] Petrovic Fabijan A., Iredell J., Danis-Wlodarczyk K., Kebriaei R., Abedon S.T. (2023). Translating phage therapy into the clinic: Recent accomplishments but continuing challenges. PLoS Biol..

[18] El-Shibiny A., El-Sahhar S. (2017). Bacteriophages: The possible solution to treat infections caused by pathogenic bacteria. Can. J. Microbiol..

[19] Kiani A.K., Anpilogov K., Dhuli K., Paolacci S., Benedetti S., Manara E., Guerri G., Dautaj A., Beccari T., Dundar M. (2021). Naturally-occurring and cultured bacteriophages in human therapy. Eur. Rev. Med. Pharmacol. Sci..

[20] Kassa T. (2021). Bacteriophages against pathogenic bacteria and possibilities for future application in Africa. Infect. Drug Resist..

[21] Ricke S.C., Lee S.I., Kim S.A., Park S.H., Shi Z. (2020). Prebiotics and the poultry gastrointestinal tract microbiome. Poult. Sci..

[22] Desiree K., Mosimann S., Ebner P. (2021). Efficacy of phage therapy in pigs: Systematic review and meta-analysis. J. Anim. Sci..

[23] Pereira A.O., Barros N.M.A., Guerrero B.R., Emencheta S.C., Baldo D.Â., Oliveira J.M., Vila M.M.D.C., Balcão V.M. (2023). An edible biopolymeric microcapsular wrapping integrating lytic bacteriophage particles for *Salmonella enterica*: Potential for integration into poultry feed. Antibiotics.

[24] Wahab A.A.-E., Basiouni S., El-Seedi H.R., Ahmed M.F.E., Bielke L.R., Hargis B., Tellez-Isaias G., Eisenreich W., Lehnherr H., Kittler S. (2023). An overview of the use of bacteriophages in the poultry industry: Successes, challenges, and possibilities for overcoming breakdowns. Front. Microbiol..

[25] Jamal M., Bukhari S.M., Andleeb S., Ali M., Raza S., Nawaz M.A., Hussain T., Rahman S.U., Shah S.S. (2019). Bacteriophages: An overview of the control strategies against multiple bacterial infections in different fields. J. Basic Microbiol..

[26] Pan L., Li D., Lin W., Liu W., Qu C., Qian M., Cai R., Zhou Q., Wang F., Tong Y. (2022). Novel aeromonas phage ahy-yong1 and its protective effects against *Aeromonas hydrophila* in brocade carp (*Cyprinus aka Koi*). Viruses.

[27] Gigante A., Atterbury R.J. (2019). Veterinary use of bacteriophage therapy in intensively-reared livestock. Virol. J..

[28] Upadhaya S.D., Ahn J.M., Cho J.H., Kim J.Y., Kang D.K., Kim S.W., Kim H.B., Kim I.H. (2021). Bacteriophage cocktail supplementation improves growth performance, gut microbiome and production traits in broiler chickens. J. Anim. Sci. Biotechnol..

[29] Papaianni M., Paris D., Woo S.L., Fulgione A., Rigano M.M., Parrilli E., Tutino M.L., Marra R., Manganiello G., Casillo A. (2020). Plant dynamic metabolic response to bacteriophage treatment after *Xanthomonas campestris* pv. *campestris* infection. Front. Microbiol..

[30] Buttimer C., McAuliffe O., Ross R.P., Hill C., O’Mahony J., Coffey A. (2017). Bacteriophages and bacterial plant diseases. Front. Microbiol..

[31] Vu N.T., Oh C.S. (2020). Bacteriophage usage for bacterial disease management and diagnosis in plants. Plant Pathol. J..

[32] Korniienko N., Kharina A., Budzanivska I., Burketová L., Kalachova T. (2022). Phages of phytopathogenic bacteria: High potential, but challenging application. Plant Prot. Sci..

[33] Holtappels D., Fortuna K., Lavigne R., Wagemans J. (2021). The future of phage biocontrol in integrated plant protection for sustainable crop production. Curr. Opin. Biotechnol..

[34] Balcão V.M., Vila M.M. (2015). Structural and functional stabilization of protein entities: State-of-the-art. Adv. Drug Deliv. Rev..

[35] Thung T.Y., Lee E., Premarathne J.M.K.J.K., Nurzafirah M., Kuan C.H., Elexson N., Tan C.W., Malcolm T.T.H., New C.Y., Ramzi O.S.B. (2018). Bacteriophages and their applications. Food Res..

[36] Cristobal-Cueto P., García-Quintanilla A., Esteban J., García-Quintanilla M. (2021). Phages in food industry biocontrol and bioremediation. Antibiotics.

[37] Van Belleghem J.D., Manasherob R., Miȩdzybrodzki R., Rogóż P., Górski A., Suh G.A., Bollyky P.L., Amanatullah D.F. (2020). The rationale for using bacteriophage to treat and prevent periprosthetic joint infections. Front. Microbiol..

[38] Altamirano F.L.G., Barr J.J. (2019). Phage Therapy in the postantibiotic era. Clin. Microbiol. Rev..

[39] Abedon S.T. (2017). Information phage therapy research should report. Pharmaceuticals.

[40] Stenberg J.A., Sundh I., Becher P.G., Björkman C., Dubey M., Egan P.A., Friberg H., Gil J.F., Jensen D.F., Jonsson M. (2021). When is it biological control? A framework of definitions, mechanisms, and classifications. J. Pest Sci..

[41] Vikram A., Woolston J., Sulakvelidze A. (2021). Phage biocontrol applications in food production and processing. Curr. Issues Mol. Biol..

[42] Rotman S.G., Sumrall E., Ziadlou R., Grijpma D.W., Richards R.G., Eglin D., Moriarty T.F. (2020). Local Bacteriophage Delivery for Treatment and Prevention of Bacterial Infections. Front. Microbiol..

[43] Malik D.J., Sokolov I.J., Vinner G.K., Mancuso F., Cinquerrui S., Vladisavljevic G.T., Clokie M.R.J., Garton N.J., Stapley A.G.F., Kirpichnikova A. (2017). Formulation, stabilization and encapsulation of bacteriophage for phage therapy. Adv. Colloid Interface Sci..

[44] Lin J., Du F., Long M., Li P. (2022). Limitations of phage therapy and corresponding optimization strategies: A review. Molecules.

[45] Loh B., Gondil V.S., Manohar P., Khan F.M., Yang H., Leptihn S. (2021). Encapsulation and delivery of therapeutic phages. Appl. Environ. Microbiol..

[46] Singla S., Harjai K., Katare O.P., Chhibber S. (2015). Bacteriophage-loaded nanostructured lipid carrier: Improved pharmacokinetics mediates effective resolution of Klebsiella pneumoniae-induced lobar pneumonia. J. Infect. Dis..

[47] Singla S., Harjai K., Katare O.P., Chhibber S. (2016). Encapsulation of bacteriophage in liposome accentuates its entry in to macrophage and shields it from neutralizing antibodies. PLoS ONE.

[48] Kiros A., Gashaw T., Teshale A. (2016). Phage therapy: A review on the biology and therapeutic application of bacteriophage. ARC J. Anim. Vet. Sci..

[49] Briot T., Kolenda C., Ferry T., Medina M., Laurent F., Leboucher G., Pirot F., PHAGEinLYON study group (2022). Paving the way for phage therapy using novel drug delivery approaches. J. Control. Release.

[50] Gill J.J., Hyman P. (2010). Phage choice, isolation, and preparation for phage therapy. Curr. Pharm. Biotechnol..

[51] Abedon S.T., Medina C., López-Baena F. (2018). Phage therapy: Various perspectives on how to improve the Art. Host-Pathogen Interactions. Methods in Molecular Biology.

[52] Baldelli A., Liang M. (2023). Design of respirable sprayed microparticles of encapsulated bacteriophages. Front. Drug Deliv..

[53] Raza A., Jamil M., Aleem M.T., Aslam M.A., Ali H.M., Khan S., Kareem N., Asghar T., Gul K., Nadeem H. (2021). Bacteriophage therapy: Recent development and applications. Sch. Bull..

[54] Ganeshan S.D., Hosseinidoust Z. (2019). Phage therapy with a focus on the human microbiota. Antibiotics.

[55] Strathdee S.A., Hatfull G.F., Mutalik V.K., Schooley R.T. (2023). Phage therapy: From biological mechanisms to future directions. Cell.

[56] Hibstu Z., Belew H., Akelew Y., Mengist H.M. (2022). Phage therapy: A different approach to fight bacterial infections. Biologics.

[57] Pardo-Freire M., Domingo-Calap P. (2023). Phages and nanotechnology: New insights against multidrug-resistant bacteria. Biodes. Res..

[58] Baldelli A., Cidem A., Guo Y., Ong H.X., Singh A., Traini D., Pratap-Singh A. (2023). Spray freeze drying for protein encapsulation: Impact of the formulation to morphology and stability. Dry. Technol..

[59] Qadir M.I., Mobeen T., Masood A. (2018). Phage therapy: Progress in pharmacokinetics. Braz. J. Pharm. Sci..

[60] Düzgüneş N., Sessevmez M., Yildirim M. (2021). Bacteriophage therapy of bacterial infections: The rediscovered frontier. Pharmaceuticals.

[61] Plumet L., Ahmad-Mansour N., Dunyach-Remy C., Kissa K., Sotto A., Lavigne J.P., Costechareyre D., Molle V. (2022). Bacteriophage therapy for Staphylococcus aureus infections: A review of animal models, treatments, and clinical trials. Front. Cell. Infect. Microbiol..

[62] Pinto A.M., Silva M.D., Pastrana L.M., Bañobre-López M., Sillankorva S. (2021). The clinical path to deliver encapsulated phages and lysins. FEMS Microbiol. Rev..

[63] Sarker S.A., Sultana S., Reuteler G., Moine D., Descombes P., Charton F., Bourdin G., McCallin S., Ngom-Bru C., Neville T. (2016). Oral phage therapy of acute bacterial diarrhea with two coliphage preparations: A randomized trial in children from Bangladesh. EBioMedicine.

[64] Choińska-Pulit A., Mituła P., Śliwka P., Łaba W., Skaradzińska A. (2015). Bacteriophage encapsulation: Trends and potential applications. Trends Food Sci.Technol..

[65] Boggione D.M.G., Batalha L.S., Gontijo M.T.P., Lopez M.E.S., Teixeira A.V.N.C., Santos I.J.B., Mendonça R.C.S. (2017). Evaluation of microencapsulation of the UFV-AREG1 bacteriophage in alginate-Ca microcapsules using microfluidic devices. Colloids Surf. B Biointerfaces.

[66] Rosner D., Clark J. (2021). Formulations for bacteriophage therapy and the potential uses of immobilization. Pharmaceuticals.

[67] Richards K., Malik D.J. (2021). Microencapsulation of bacteriophages using membrane emulsification in different pH-triggered controlled release formulations for oral administration. Pharmaceuticals.

[68] Gkartziou F., Giormezis N., Spiliopoulou I., Antimisiaris S.G. (2021). Nanobiosystems for antimicrobial drug-resistant infections. Nanomaterials.

[69] Devi C.B.P., Harshitha B., Deekshitha S., Sudha Rani G., Sharma J.V.C. (2019). Phage therapy—A new weapon to fight against the superbugs. Int. J. Res. Anal. Rev..

[70] Musin E.V., Kim A.L., Dubrovskii A.V., Kudryashova E.B., Ariskina E.V., Tikhonenko S.A. (2021). The influence of polyanions and polycations on bacteriophage activity. Polymers.

[71] Schubert C., Fischer S., Dorsch K., Teßmer L., Hinrichs J., Atamer Z. (2022). Microencapsulation of bacteriophages for the delivery to and modulation of the human gut microbiota through milk and cereal products. Appl. Sci..

[72] Rahimzadeh G., Saeedi M., Moosazadeh M., Hashemi S.M.H., Babaei A., Rezai M.S., Kamel K., Asare-Addo K., Nokhodchi A. (2021). Encapsulation of bacteriophage cocktail into chitosan for the treatment of bacterial diarrhea. Sci. Rep..

[73] Musin E.V., Kim A.L., Dubrovskii A.V., Ariskina E.V., Kudryashova E.B., Tikhonenko S.A. (2022). The pathways to create containers for bacteriophage delivery. Polymers.

[74] Dini C., Islan G.A., Castro G.R. (2014). Characterization and stability analysis of biopolymeric matrices designed for phage-controlled release. Appl. Biochem. Biotechnol..

[75] Ergin F., Atamer Z., Göcer E.M.C., Demir M., Hinrichs J., Kucukcetin A. (2021). Optimization of bacteriophage microencapsulation in alginate-caseinate formulation using vibrational nozzle technique. Food Hydrocoll..

[76] Vinner G.K., Vladisavljević G.T., Clokie M.R.J., Malik D.J. (2017). Microencapsulation of *Clostridium difficile* specific bacteriophages using microfluidic glass capillary devices for colon delivery using pH triggered release. PLoS ONE.

[77] Vinner G.K., Richards K., Leppanen M., Sagona A.P., Malik D.J. (2019). Microencapsulation of enteric bacteriophages in a pH-responsive solid oral dosage formulation using a scalable membrane emulsification process. Pharmaceutics.

[78] Vinner G.K., Malik D.J. (2018). High precision microfluidic microencapsulation of bacteriophages for enteric delivery. Res. Microbiol..

[79] Malik D.J., Faintuch J., Faintuch S. (2019). Chapter 19—Targeted delivery of bacteriophages to the gastrointestinal tract and their controlled release: Unleashing the therapeutic potential of phage therapy. Microbiome and Metabolome in Diagnosis, Therapy, and Other Strategic Applications.

[80] Śliwka P., Mituła P., Mituła A., Skaradziński G., Choińska-Pulit A., Niezgoda N., Weber-Dąbrowska B., Żaczek M., Skaradzińska A. (2019). Encapsulation of bacteriophage T4 in mannitol-alginate dry macrospheres and survival in simulated gastrointestinal conditions. LWT—Food Sci. Technol..

[81] Kim S., Jo A., Ahn J. (2015). Application of chitosan-alginate microspheres for the sustained release of bacteriophage in simulated gastrointestinal conditions. Int. J. Food Sci. Technol..

[82] Moghtader F., Eğri S., Piskin E. (2017). Phages in modified alginate beads. Artif. Cells Nanomed. Biotechnol..

[83] Abdelsattar A.S., Abdelrahman F., Dawoud A., Connerton I.F., El-Shibiny A. (2019). Encapsulation of E. coli phage ZCEC5 in chitosan-alginate beads as a delivery system in phage therapy. AMB Express..

[84] Nieth A., Verseux C., Barnert S., Süss R., Römer W. (2015). A first step toward liposome-mediated intracellular bacteriophage therapy. Expert Opin. Drug Deliv..

[85] Chhibber S., Kaur J., Kaur S. (2018). Liposome entrapment of bacteriophages improves wound healing in a diabetic mouse MRSA infection. Front. Microbiol..

[86] Cinquerrui S., Mancuso F., Vladisavljević G.T., Bakker S.E., Malik D.J. (2018). Nanoencapsulation of bacteriophages in liposomes prepared using microfluidic hydrodynamic flow focusing. Front. Microbiol..

[87] Cafora M., Poerio N., Forti F., Loberto N., Pin D., Bassi R., Aureli M., Briani F., Pistocchi A., Fraziano M. (2022). Evaluation of phages and liposomes as combination therapy to *counteract Pseudomonas aeruginosa* infection in wild-type and CFTR-null models. Front. Microbiol..

[88] Otero J., García-Rodríguez A., Cano-Sarabia M., Maspoch D., Marcos R., Cortés P., Llagostera M. (2019). Biodistribution of liposome-encapsulated bacteriophages and their transcytosis during oral phage therapy. Front. Microbiol..

[89] Jung D., Gaudreau-Lapierre A., Alnahhas E., Asraoui S. (2021). Bacteriophage-liposomes complex, a bi-therapy system to target *Streptococcus pneumonia* and biofilm: A research protocol. Undergrad. Res. Nat. Clin. Sci. Technol. J..

[90] Kopač T., Lisac A., Mravljak R., Ručigaj A., Krajnc M., Podgornik A. (2021). Bacteriophage delivery systems based on composite polyHIPE/nanocellulose hydrogel particles. Polymers.

[91] Chadha P., Katare O.P., Chhibber S. (2017). Liposome loaded phage cocktail: Enhanced therapeutic potential in resolving *Klebsiella pneumoniae* mediated burn wound infections. Burns.

[92] Steele A., Stacey H.J., de Soir S., Jones J.D. (2020). The safety and efficacy of phage therapy for superficial bacterial infections: A systematic review. Antibiotics.

[93] Durr H.A., Leipzig N.D. (2023). Advancements in bacteriophage therapies and delivery for bacterial infection. Mater. Adv..

[94] Ferry T., Leboucher G., Fevre C., Herry Y., Conrad A., Josse J., Batailler C., Chidiac C., Medina M., Lustig S. (2018). Salvage debridement, antibiotics and implant retention (“DAIR”) with local injection of a selected cocktail of bacteriophages: Is it an option for an elderly patient with relapsing *Staphylococcus aureus* prosthetic-joint infection?. Open Forum Infect. Dis..

[95] Tkhilaishvili T., Winkler T., Müller M., Perka C., Trampuz A. (2019). Bacteriophages as adjuvant to antibiotics for the treatment of periprosthetic joint infection caused by multidrug-resistant *Pseudomonas aeruginosa*. Antimicrob. Agents Chemother..

[96] Gupta P., Singh H.S., Shukla V.K., Nath G., Bhartiya S.K. (2019). Bacteriophage therapy of chronic nonhealing wound: Clinical study. Int. J. Low Extrem. Wounds.

[97] Pinto A.M., Cerqueira M.A., Bañobre-Lópes M., Pastrana L.M., Sillankorva S. (2020). Bacteriophages for chronic wound treatment: From traditional to novel delivery systems. Viruses.

[98] Chang R.Y.K., Morales S., Okamoto Y., Chan H.K. (2020). Topical application of bacteriophages for treatment of wound infections. Transl. Res..

[99] Brown T.L., Petrovski S., Chan H.T., Angove M.J., Tucci J. (2018). Semi-solid and solid dosage forms for the delivery of phage therapy to epithelia. Pharmaceuticals.

[100] Aghaee B.L., Khan Mirzaei M., Alikhani M.Y., Mojtahedi A., Maurice C.F. (2021). Improving the inhibitory effect of phages against *Pseudomonas aeruginosa* isolated from a burn patient using a combination of phages and antibiotics. Viruses.

[101] Golembo M., Puttagunta S., Rappo U., Weinstock E., Engelstein R., Gahali-Sass I., Moses A., Kario E., Ben-Dor Cohen E., Nicenboim J. (2022). Development of a topical bacteriophage gel targeting *Cutibacterium acnes* for acne prone skin and results of a phase 1 cosmetic randomized clinical trial. Skin Health Dis..

[102] Balcão V.M., Glasser C.A., Chaud M.V., del Fiol F.S., Tubino M., Vila M.M. (2014). Biomimetic aqueous-core lipid nanoballoons integrating a multiple emulsion formulation: A suitable housing system for viable lytic bacteriophages. Colloids Surf. B Biointerfaces.

[103] Beschastnov V.V., Ryabkov M.G., Leontiev A.E., Tulupov A.A., Yudanova T.N., Shirokova I.Y., Belyanina N.A., Kovalishena O.V. (2021). Viability of bacteriophages in the complex hydrogel wound dressings in vitro. Sovrem. Tekhnologii. Med..

[104] Batinovic S., Wassef F., Knowler S.A., Rice D.T.F., Stanton C.R., Rose J., Tucci J., Nittami T., Vinh A., Drummond G.R. (2019). Bacteriophages in natural and artificial environments. Pathogens.

[105] Campos W.F., Silva E.C., Oliveira T.J., Oliveira J.M., Tubino M., Pereira C., Vila M.M., Balcão V.M. (2020). Transdermal permeation of bacteriophage particles by choline oleate: Potential for treatment of soft-tissue infections. Future Microbiol..

[106] Li J., Mooney D. (2016). Designing hydrogels for controlled drug delivery. Nat. Rev. Mater..

[107] Khan S., Ullah A., Ullah K., Rehman N.-U. (2016). Insight into hydrogels. Des. Monomers Polym..

[108] Chang RY K., Okamoto Y., Morales S., Kutter E., Chan H.K. (2021). Hydrogel formulations containing non-ionic polymers for topical delivery of bacteriophages. Int. J. Pharm..

[109] Merabishvili M., Monserez R., van Belleghem J., Rose T., Jennes S., De Vos D., Verbeken G., Vaneechoutte M., Pirnay J.P. (2017). Stability of bacteriophages in burn wound care products. PLoS ONE.

[110] Nogueira F., Karumidze N., Kusradze I., Goderdzishvili M., Teixeira P., Gouveia I.C. (2017). Immobilization of bacteriophage in wound-dressing nanostructure. Nanomedicine.

[111] Kaur P., Gondil V.S., Chhibber S. (2019). A novel wound dressing consisting of PVA-SA hybrid hydrogel membrane for topical delivery of bacteriophages and antibiotics. Int. J. Pharm..

[112] Boggione D.M.G., Santos I.J.B., de Souza S.M., Mendonça R.C.S. (2021). Preparation of polyvinyl alcohol hydrogel containing bacteriophage and its evaluation for potential use in the healing of skin wounds. J. Drug Deliv. Sci Technol..

[113] Zhang J., Ge J., Xu Y., Chen J., Zhou A., Sun L., Gao Y., Zhang Y., Gu T., Ning X. (2020). Bioactive multi-engineered hydrogel offers simultaneous promise against antibiotic resistance and wound damage. Int. J. Biol. Macromol..

[114] Veverka M., Dubaj T., Gallovič J., Veverková E., Šimon P., Lokaj J., Jorík V. (2020). Formulations of Staphylococcus aureus bacteriophage in biodegradable beta-glucan and arabinogalactan-based matrices. J. Drug Deliv. Sci. Technol..

[115] Barros J.A.R., Melo L.D.R., Silva R.A.R.D., Ferraz M.P., Azeredo J.C.V.R., Pinheiro V.M.C., Colaço B.J.A., Fernandes M.H.R., Gomes P.S., Monteiro F.J. (2020). Encapsulated bacteriophages in alginate-nanohydroxyapatite hydrogel as a novel delivery system to prevent orthopedic implant-associated infections. Nanomedicine.

[116] Tkhilaishvili T., Merabishvili M., Pirnay J.P., Starck C., Potapov E., Falk V., Schoenrath F. (2021). Successful case of adjunctive intravenous bacteriophage therapy to treat left ventricular assist device infection. J. Infect..

[117] Speck P., Smithyman A. (2016). Safety and efficacy of phage therapy via the intravenous route. FEMS Microbiol. Lett..

[118] Aslam S., Lampley E., Wooten D., Karris M., Benson C., Strathdee S., Schooley R.T. (2020). Lessons learned from the first 10 consecutive cases of intravenous bacteriophage therapy to treat multidrug-resistant bacterial infections at a single center in the United States. Open Forum Infect. Dis..

[119] Aslam S. (2020). Bacteriophage therapy as a treatment option for transplant infections. Curr. Opin. Infect. Dis..

[120] Ali Y., Inusa I., Sanghvi G., Mandaliya V.B., Bishoyi A.K. (2023). The current status of phage therapy and its advancement towards establishing standard antimicrobials for combating multi drug-resistant bacterial pathogens. Microb. Pathog..

[121] Doub J.B., Ng V.Y., Johnson A.J., Slomka M., Fackler J., Horne B., Brownstein M.J., Henry M., Malagon F., Biswas B. (2020). Salvage bacteriophage therapy for a chronic MRSA prosthetic joint infection. Antibiotics.

[122] Rao S., Betancourt-Garcia M., Kare-Opaneye Y.O., Swierczewski B.E., Bennett J.W., Horne B., Fackler J., Suazo Hernandez L.P., Brownstein M.J. (2022). Critically Ill patient with multidrug-resistant *Acinetobacter baumannii* respiratory infection successfully treated with intravenous and nebulized bacteriophage therapy. Antimicrob. Agents Chemother..

[123] Law N., Logan C., Yung G., Furr C.L., Lehman S.M., Morales S., Rosas F., Gaidamaka A., Bilinsky I., Grint P. (2019). Successful adjunctive use of bacteriophage therapy for treatment of multidrug-resistant Pseudomonas aeruginosa infection in a cystic fibrosis patient. Infection.

[124] Schoeffel J., Wang E.W., Gill D., Frackler J., Horne B., Manson T., Doub J.B. (2022). Successful use of salvage bacteriophage therapy for a recalcitrant MRSA knee and hip prosthetic joint infection. Pharmaceuticals.

[125] Ramirez-Sanchez C., Gonzales F., Buckley M., Biswas B., Henry M., Deschenes M.V., Horne B., Fackler J., Brownstein M.J., Schooley R.T. (2021). Successful treatment of *Staphylococcus aureus* prosthetic joint infection with bacteriophage therapy. Viruses.

[126] Aslam S., Pretorius V., Adler E., Lehman S., Morales S., Gaidamaka A., Furr C., Rosas F., Bishop-Lilly K., Biswas B. (2019). Novel bacteriophage therapy for treatment of ventricular assist device infections. J. Heart Lung Transplant..

[127] Ferry T., Kolenda C., Laurent F., Leboucher G., Merabischvilli M., Djebara S., Gustave C.A., Perpoint T., Barrey C., Pirnay J.P. (2022). Personalized bacteriophage therapy to treat pandrug-resistant spinal Pseudomonas aeruginosa infection. Nat. Commun..

[128] Schooley R.T., Biswas B., Gill J.J., Hernandez-Morales A., Lancaster J., Lessor L., Barr J.J., Reed S.L., Rohwer F., Benler S. (2017). Development and use of personalized bacteriophage-based therapeutic cocktails to treat a patient with a disseminated resistant *Acinetobacter baumannii* infection. Antimicrob. Agents Chemother..

[129] Chang RY K., Wallin M., Lin Y., Leung S.S.Y., Wang H., Morales S., Chan H.K. (2018). Phage therapy for respiratory infections. Adv. Drug Deliv. Rev..

[130] Hoe S., Semler D.D., Goudie A.D., Lynch K.H., Matinkhoo S., Finlay W.H., Dennis J.J., Vehring R. (2013). Respirable bacteriophages for the treatment of bacterial lung infections. J. Aerosol Med. Pulm. Drug Deliv..

[131] Iszatt J.J., Larcombe A.N., Chan H.K., Stick S.M., Garratt L.W., Kicic A. (2021). Phage therapy for multi-drug resistant respiratory tract infections. Viruses.

[132] Porat S.B., Gelman D., Yerushalmy O., Alkalay-Oren S., Coppenhagen-Glazer S., Cohen-Cymberknoh M., Kerem E., Amirav I., Nir-Paz R., Hazan R. (2021). Expanding clinical phage microbiology: Simulating phage inhalation for respiratory tract infections. ERJ Open Res..

[133] Rios A.C., Vila M.M.D.C., Lima R., Del Fiol F.S., Tubino M., Teixeira J.A., Balcão V.M. (2018). Structural and functional stabilization of bacteriophage particles within the aqueous core of a W/O/W multiple emulsion: A potential biotherapeutic system for the inhalational treatment of bacterial pneumonia. Process Biochem..

[134] Valente L.G., Federer L., Iten M., Grandgirard D., Leib S.L., Jakob S.M., Haenggi M., Cameron D.R., Que Y.A., Prazak J. (2021). Searching for synergy: Combining systemic daptomycin treatment with localized phage therapy for the treatment of experimental pneumonia due to MRSA. BMC Res. Notes.

[135] Speck P.G., Warner M.S., Bihari S., Bersten A.D., Mitchell J.G., Tucci J., Gordon D.L. (2021). Potential for bacteriophage therapy for Staphylococcus aureus pneumonia with influenza A coinfection. Future Microbiol..

[136] Nick J.A., Dedrick R.M., Gray A.L., Vladar E.K., Smith B.E., Freeman K.G., Malcolm K.C., Epperson L.E., Hasan N.A., Hendrix J. (2022). Host and pathogen response to bacteriophage engineered against *Mycobacterium abscessus* lung infection. Cell.

[137] Lebeaux D., Merabishvili M., Caudron E., Lannoy D., Van Simaey L., Duyvejonck H., Guillemain R., Thumerelle C., Podglajen I., Compain F. (2021). A case of phage therapy against pandrug-resistant *Achromobacter xylosoxidans* in a 12-year-old lung-transplanted cystic fibrosis patient. Viruses.

[138] Singh J., Fitzgerald D.A., Jaffe A., Hunt S., Barr J.J., Iredell J., Selvadurai H. (2023). Single-arm, open-labelled, safety and tolerability of intrabronchial and nebulised bacteriophage treatment in children with cystic fibrosis and Pseudomonas aeruginosa. BMJ Open Resp. Res..

[139] Naureen Z., Malacarne D., Anpilogov K., Dautaj A., Camilleri G., Cecchin S., Bressan S., Casadei A., Albion E., Sorrentino E. (2020). Comparison between American and European legislation in the therapeutical and alimentary bacteriophage usage. Acta Biomed..

[140] Merabishvili M., Pirnay J.P., Vogele K., Malik D.J., Górski A., Międzybrodzki R., Borysowski J. (2019). Production of phage therapeutics and formulations: Innovative approaches. Phage Therapy: A Practical Approach.

[141] Loponte R., Pagnini U., Iovane G., Pisanelli G. (2021). Phage therapy in veterinary medicine. Antibiotics.

[142] Pyzik E., Radzki R.P., Urban-Chmiel R. (2021). experimental phage therapies in companion animals with a historical review. Curr. Rev. Clin. Exp. Pharmacol..

[143] Squires R.A. (2021). Bacteriophage therapy for challenging bacterial infections: Achievements, limitations and prospects for future clinical use by veterinary dermatologists. Vet. Dermatol..

[144] Ravi Y., Pooja M.K., Yadav D.K.K. (2017). Review—bacteriophages in food preservation. Int. J. Pure Appl. Biosci..

[145] Alomari M.M.M., Dec M., Urban-Chmiel R. (2021). Bacteriophages as an alternative method for control of zoonotic and foodborne pathogens. Viruses.

[146] Alomari MM M., Dec M., Nowaczek A., Puchalski A., Wernicki A., Kowalski C., Urban-Chmiel R. (2021). Therapeutic and prophylactic effect of the experimental bacteriophage treatment to control diarrhea caused by *E. coli* in Newborn Calves. ACS Infect. Dis..

[147] Kazi M., Annapure U.S. (2016). Bacteriophage biocontrol of foodborne pathogens. J. Food Sci. Technol..

[148] Colom J., Cano-Sarabia M., Otero J., Aríñez-Soriano J., Cortés P., Maspoch D., Llagostera M. (2017). Microencapsulation with alginate/CaCO_3_: A strategy for improved phage therapy. Sci. Rep..

[149] Soto M.J., Retamales J., Palza H., Bastías R. (2018). Encapsulation of specific *Salmonella enteritidis* phage f3αSE on alginate-spheres as a method for protection and dosification. Electron. J. Biotechnol..

[150] Yin H., Li J., Huang H., Wang Y., Qian X., Ren J., Xue F., Dai J., Tang F. (2021). Microencapsulated phages show prolonged stability in gastrointestinal environments and high therapeutic efficiency to treat Escherichia coli O157:H7 infection. Vet. Res..

[151] Kaikabo A.A., AbdulKarim S.M., Abas F. (2017). Evaluation of the efficacy of chitosan nanoparticles loaded ΦKAZ14 bacteriophage in the biological control of colibacillosis in chickens. Poult. Sci..

[152] Gomez-Garcia J., Chavez-Carbajal A., Segundo-Arizmendi N., Baron-Pichardo M.G., Mendoza-Elvira S.E., Hernandez-Baltazar E., Hynes A.P., Torres-Angeles O. (2021). Efficacy of salmonella bacteriophage S1 delivered and released by alginate beads in a chicken model of infection. Viruses.

[153] Lorenzo-Rebenaque L., Malik D.J., Catalá-Gregori P., Marin C., Sevilla-Navarro S. (2022). Gastrointestinal dynamics of non-encapsulated and microencapsulated salmonella bacteriophages in broiler production. Animals.

[154] Zhang B., Wang Y., Wang F., Zhang Y., Hao H., Lv X., Hao L., Shi Y. (2023). Microencapsulated phage composites with increased gastrointestinal stability for the oral treatment of Salmonella colonization in chicken. Front. Vet. Sci..

[155] Ma Y.-H., Islam G.S., Wu Y., Sabour P.M., Chambers J.R., Wang Q., Wu S.X.Y., Griffiths M.W. (2016). Temporal distribution of encapsulated bacteriophages during passage through the chick gastrointestinal tract. Poult. Sci..

[156] Chen B., Benavente L.P., Chittò M., Wychowaniec J.K., Post V., D’Este M., Constant C., Zeiter S., Feng W., Moreno M.G. (2023). Alginate microbeads and hydrogels delivering meropenem and bacteriophages to treat Pseudomonas aeruginosa fracture-related infections. J. Control. Release.

[157] Zhu T., Yang C., Bao X., Chen F., Guo X. (2022). Strategies for controlling biofilm formation in food industry. Grain Oil Sci. Technol..

[158] Hernández I. (2017). Bacteriophages against serratia as fish spoilage control technology. Front. Microbiol..

[159] Moye Z.D., Woolston J., Sulakvelidze A. (2018). Bacteriophage applications for food production and processing. Viruses.

[160] Wójcik E.A., Stańczyk M., Wojtasik A., Kowalska J.D., Nowakowska M., Łukasiak M., Bartnicka M., Kazimierczak J., Dastych J. (2020). Comprehensive evaluation of the safety and efficacy of BAFASAL^®^ bacteriophage preparation for the reduction of salmonella in the food chain. Viruses.

[161] Iacumin L., Manzano M., Comi G. (2016). Phage inactivation of *Listeria monocytogenes* on San Daniele dry-cured ham and elimination of biofilms from equipment and working environments. Microorganisms.

[162] Ferriol-González C., Domingo-Calap P. (2021). Phage therapy in livestock and companion animals. Antibiotics.

[163] Keerqin C., McGlashan K., Van T.T.H., Chinivasagam H.N., Moore R.J., Choct M., Wu S.B. (2022). A lytic bacteriophage isolate reduced *Clostridium perfringens* induced lesions in necrotic enteritis challenged broilers. Front. Vet. Sci..

[164] Pelyuntha W., Yafa A., Ngasaman R., Yingkajorn M., Chukiatsiri K., Champoochana N., Vongkamjan K. (2022). Oral administration of a phage cocktail to reduce salmonella colonization in broiler gastrointestinal tract—A pilot study. Animals.

[165] Kosznik-Kwaśnicka K., Podlacha M., Grabowski Ł., Stasiłojć M., Nowak-Zaleska A., Ciemińska K., Cyske Z., Dydecka A., Gaffke L., Mantej J. (2022). Biological aspects of phage therapy versus antibiotics against *Salmonella enterica* serovar *Typhimurium* infection of chickens. Front. Cell. Infect. Microbiol..

[166] Clavijo V., Baquero D., Hernandez S., Farfan J.C., Arias J., Arévalo A., Donado-Godoy P., Vives-Flores M. (2019). Phage cocktail SalmoFREE^®^ reduces Salmonella on a commercial broiler farm. Poult. Sci..

[167] Ninawe A.S., Sivasankari S., Ramasamy P., Seghal Kiran G., Selvin J. (2020). Bacteriophages for aquaculture disease control. Aquacult. Int..

[168] Kunttu HM T., Runtuvuori-Salmela A., Middelboe M., Clark J., Sundberg L.R. (2021). Comparison of delivery methods in phage therapy against *Flavobacterium columnare* infections in rainbow trout. Antibiotics.

[169] Schulz P., Pajdak-Czaus J., Siwicki A.K. (2022). In vivo bacteriophages’ application for the prevention and therapy of aquaculture animals-chosen aspects. Animals.

[170] Choudhury T.G., Nagaraju V.T., Gita S., Paria A., Parhi J. (2017). Advances in Bacteriophage research for bacterial disease control in aquaculture. Rev. Fish. Sci. Aquac..

[171] Hawkins C., Harper D., Burch D., Anggård E., Soothill J. (2010). Topical treatment of *Pseudomonas aeruginosa* otitis of dogs with a bacteriophage mixture: A before/after clinical trial. Vet. Microbiol..

[172] Silva E.C., Oliveira T.J., Moreli F.C., Harada L.K., Vila M.M.D.C., Balcão V.M. (2021). Newly isolated lytic bacteriophages for *Staphylococcus intermedius*, structurally and functionally stabilized in a hydroxyethylcellulose gel containing choline geranate: Potential for transdermal permeation in veterinary phage therapy. Res. Vet. Sci..

[173] Solomon S.E.B., de Farias M.R., Pimpão C.T. (2016). Use of *Staphylococcus aureus* Phage Lysate Staphage Lysate (SPL)^®^ for the control of recurrent pyoderma eczema in dogs with atopic dermatitis. Acta Sci. Vet..

[174] Balcão V.M., Belline B.G., Silva E.C., Almeida P.F.F.B., Baldo D.Â., Amorim L.R.P., Oliveira Júnior J.M., Vila M.M.D.C., Del Fiol F.S. (2022). Isolation and molecular characterization of two novel lytic bacteriophages for the biocontrol of Escherichia coli in uterine infections: In vitro and ex vivo preliminary studies in veterinary medicine. Pharmaceutics.

[175] Ioannou P., Baliou S., Samonis G. (2023). Bacteriophages in infectious diseases and beyond—A narrative review. Antibiotics.

[176] Pereira C., Costa P., Pinheiro L., Balcão V.M., Almeida A. (2021). Kiwifruit bacterial canker: An integrative view focused on biocontrol strategies. Planta.

[177] Villalpando-Aguilar J.L., Matos-Pech G., López-Rosas I., Castelán-Sánchez H.G., Alatorre-Cobos F. (2022). Phage therapy for crops: Concepts, experimental and bioinformatics approaches to direct its application. Int. J. Mol. Sci..

[178] Farooq T., Hussain M.D., Shakeel M.T., Tariqjaveed M., Aslam M.N., Naqvi S.A.H., Amjad R., Tang Y., She X., He Z. (2022). Deploying viruses against phytobacteria: Potential use of phage cocktails as a multifaceted approach to combat resistant bacterial plant pathogens. Viruses.

[179] Baliyan N., Dhiman S., Dheeman S., Vishnoi V.K., Kumar S., Maheshwari D.K. (2022). Bacteriophage cocktails as antibacterial agents in crop protection. Environ. Sustain..

[180] Laglaguano J.C., Cordova A.V. (2019). Bacteriophages applications in agriculture. Lat. Am. J. Biotechnol. Life Sci.—Bionatura Conf. Ser..

[181] Ibrahim Y.E., Saleh A.A., Al-Saleh M.A. (2017). Management of Asiatic citrus canker under field conditions in Saudi Arabia using bacteriophages and acibenzolar-s-methyl. Plant Dis..

[182] Bae J.Y., Wu J., Lee H.J., Jo E.J., Murugaiyan S., Chung E., Lee S.W. (2012). Biocontrol potential of a lytic bacteriophage PE204 against bacterial wilt of tomato. J. Microbiol. Biotechnol..

[183] Wei C., Liu J., Maina A.N., Mwaura F.B., Yu J., Yan C., Zhang R., Wei H. (2017). Developing a bacteriophage cocktail for biocontrol of potato bacterial wilt. Virol. Sin..

[184] Mousa S., Magdy M., Xiong D., Nyaruabaa R., Rizk S.M., Yu J., Wei H. (2022). Microbial profiling of potato-associated rhizosphere bacteria under bacteriophage therapy. Antibiotics.

[185] Tarakanov R.I., Lukianova A.A., Evseev P.V., Pilik R.I., Tokmakova A.D., Kulikov E.E., Toshchakov S.V., Ignatov A.N., Dzhalilov F.S., Miroshnikov K.A. (2022). Ayka, a novel curtobacterium bacteriophage, provides protection against soybean bacterial wilt and tan spot. Int. J. Mol. Sci..

[186] Choe J., Kim B., Park M.K., Roh E. (2023). Biological and genetic characterizations of a novel lytic ΦFifi106 against indigenous *Erwinia amylovora* and evaluation of the control of fire blight in apple plants. Biology.

[187] Sousa D.M., Janssen L., Rosa R.B., Belmok A., Yamada J.K., Corrêa R.F.T., de Souza Andrade M., Inoue-Nagata A.K., Ribeiro B.M., de Carvalho Pontes N. (2023). Isolation, characterization, and evaluation of putative new bacteriophages for controlling bacterial spot on tomato in Brazil. Arch. Virol..

[188] Skliros D., Papazoglou P., Gkizi D., Paraskevopoulou E., Katharios P., Goumas D.E., Tjamos S., Flemetakis E. (2023). In planta interactions of a novel bacteriophage against *Pseudomonas syringae* pv. *tomato*. Appl. Microbiol. Biotechnol..

[189] Petrzik K., Vacek J., Kmoch M., Binderová D., Brázdová S., Lenz O., Ševčík R. (2023). Field use of protective bacteriophages against pectinolytic bacteria of potato. Microorganisms.

[190] Kaur S., Kumari A., Kumari Negi A., Galav V., Thakur S., Agrawal M., Sharma V. (2021). Nanotechnology based Approaches in phage therapy: Overcoming the pharmacological barriers. Front. Pharmacol..

[191] Ranjani P., Gowthami Y., Gnanamanickam S.S., Palani P. (2018). Bacteriophages: A new weapon for the control of bacterial blight disease in rice caused by *Xanthomonas oryzae*. Microbiol. Biotechnol. Lett..

[192] Flores O., Retamales J., Núñez M., León M., Salinas P., Besoain X., Yañez C., Bastías R. (2020). Characterization of bacteriophages against *Pseudomonas syringae* pv. *actinidiae* with potential use as natural antimicrobials in kiwifruit plants. Microorganisms.

[193] Pinheiro LA M., Pereira C., Barreal M.E., Gallego P.P., Balcão V.M., Almeida A. (2019). Use of phage ϕ6 to inactivate *Pseudomonas syringae* pv. *actinidiae* in kiwifruit plants: In vitro and ex vivo experiments. Appl. Microbiol. Biotechnol..

[194] Silva E.C., Rodrigues L.M.R., Vila M.M.D.C., Balcão V.M. (2023). Newly isolated phages preying on *Pseudomonas syringae* pv. *garcae:* In vitro and ex vivo inactivation studies in coffee plant leafs. Enzyme Microb. Technol..

[195] Luo J., Dai D., Lv L., Ahmed T., Chen L., Wang Y., An Q., Sun G., Li B. (2022). Advancements in the use of bacteriophages to combat the kiwifruit canker phytopathogen *Pseudomonas syringae* pv. *actinidiae*. Viruses.

[196] Jones J.B., Vallad G.E., Iriarte F.B., Obradović A., Wernsing M.H., Jackson L.E., Balogh B., Hong J.C., Momol M.T. (2012). Considerations for using bacteriophages for plant disease control. Bacteriophage.

[197] Frampton R.A., Pitman A.R., Fineran P.C. (2012). Advances in bacteriophage-mediated control of plant pathogens. Int. J. Microbiol..

[198] Balogh B., Jones J.B., Momol M.T., Olson S.M., Obradovic A., King P., Jackson L.E. (2003). Improved efficacy of newly formulated bacteriophages for management of bacterial spot on tomato. Plant. Dis..

[199] Lang J.M., Gent D.H., Schwartz H.F. (2007). Management of xanthomonas leaf blight of onion with bacteriophages and a plant activator. Plant Dis..

[200] Gašić K., Kuzmanović N., Ivanović M., Prokić A., Šević M., Obradović A. (2018). complete genome of the *Xanthomonas euvesicatoria* specific bacteriophage KΦ1, Its survival and potential in control of pepper bacterial spot. Front. Microbiol..

[201] Karn M., Sharma S.K., Handa A., Sharma A., Sharma S., Sharma U. (2023). Lytic bacteriophages in preventing the bacterial blight of pomegranate caused by *Xanthomonas axonopodis* pv. *punicae*. Vegetos.

[202] Svircev A., Roach D., Castle A. (2018). Framing the future with bacteriophages in agriculture. Viruses.

[203] Doffkay Z., Dömötör D., Kovács T., Rákhely G. (2015). Bacteriophage therapy against plant, animal and human pathogens. Acta Biol. Szeged..

